# Type 2 Diabetes Mellitus as a Multisystem Disease: From Insulin Resistance to Organ Crosstalk—A Narrative Review

**DOI:** 10.3390/biomedicines14040752

**Published:** 2026-03-26

**Authors:** Héctor Fuentes-Barría, Raúl Aguilera-Eguía, Cherie Flores-Fernández, Lissé Angarita-Davila, Miguel Alarcón-Rivera

**Affiliations:** 1 Centro de Investigación en Medicina de Altura (CEIMA), Universidad Arturo Prat, Iquique 1110939, Chile; 2 Departamento de Salud Pública, Facultad de Medicina, Universidad Católica de la Santísima Concepcion, Concepcion 3349001, Chile; raguilerae@ucsc.cl; 3 Departamento Gestión de la Información, Universidad Tecnológica Metropolitana, Santiago 7550000, Chile; cflores@utem.cl; 4 Escuela de Nutrición y Dietética, Facultad de Medicina, Universidad Andres Bello, Concepcion 3349001, Chile; lisse.angarita@unab.cl; 5 Escuela de Ciencias del Deporte y Actividad Física, Facultad de Salud, Universidad Santo Tomas, Talca 3460000, Chile; mrivera3@santotomas.cl

**Keywords:** type 2 diabetes mellitus, molecular biology, signal transduction, glucose metabolism disorders, therapeutics

## Abstract

Type 2 Diabetes Mellitus (T2DM) is a complex metabolic disorder characterized by insulin resistance, chronic low-grade inflammation, and progressive metabolic dysfunction affecting multiple organs. This review explores the molecular and physiological mechanisms underlying T2DM, emphasizing the role of intracellular metabolic signaling pathways, mitochondrial function, and inter-organ communication in the development and progression of metabolic dysregulation. Particular attention is given to key regulatory pathways such as AMP-activated protein kinase (AMPK) and the mechanistic target of rapamycin (mTOR), which play central roles in cellular energy sensing, glucose metabolism, and lipid homeostasis. Dysregulation of these pathways contributes to impaired insulin signaling, mitochondrial dysfunction, oxidative stress, and altered adipogenesis, all of which are critical factors in the pathophysiology of T2DM. In addition, growing evidence highlights the importance of metabolic crosstalk between skeletal muscle, adipose tissue, liver, pancreas, and the gut microbiota through signaling molecules including adipokines, myokines, hepatokines, and gut-derived metabolites. These inter-organ networks influence systemic inflammation, metabolic flexibility, and glucose homeostasis. Lifestyle factors such as physical activity, nutritional patterns, and micronutrient status have also been shown to modulate these molecular pathways, improving mitochondrial function and insulin sensitivity while reducing inflammatory signaling. Despite significant advances in understanding the molecular basis of T2DM, important challenges remain, including heterogeneity in disease progression and variability in individual metabolic responses. In conclusion, T2DM should be understood as a multisystem metabolic disorder driven by complex interactions between molecular signaling pathways and systemic metabolic regulation. Future research integrating molecular mechanisms with clinical and lifestyle interventions may help develop more effective strategies for prevention and treatment.

## 1. Introduction

Type 2 diabetes mellitus (T2DM) is one of the most prevalent metabolic disorders worldwide and represents a major global health challenge [1]. According to the International Diabetes Federation, more than 589 million adults are currently living with diabetes, and this number is expected to rise substantially in the coming decades [2]. Traditionally, T2DM has been considered a disease primarily characterized by insulin resistance and pancreatic β-cell dysfunction [3,4]. In addition, pancreatic inflammation has emerged as a critical contributor to β-cell dysfunction, impairing insulin secretion and promoting β-cell apoptosis [3,4]. However, growing evidence suggests that T2DM should instead be understood as a complex multisystem disorder involving coordinated metabolic alterations across multiple organs and tissues [5,6,7,8].

In recent years, the pathophysiological understanding of T2DM has shifted from a pancreas-centric view to a more integrative model that emphasizes dynamic interactions among the liver, skeletal muscle, adipose tissue, pancreas, gut, immune system, and central nervous system [9,10]. These tissues communicate through metabolic substrates, hormones, cytokines, and signaling molecules that collectively regulate systemic glucose and lipid homeostasis [11,12,13]. Disruption of this inter-organ communication network promotes the onset of insulin resistance, chronic inflammation, mitochondrial dysfunction, and a progressive decline in metabolic function [10,12,14,15]. Within this network, pancreatic inflammation plays a key role by linking systemic inflammatory signals with local islet dysfunction, further aggravating metabolic imbalance [4].

Among the organs involved in metabolic regulation, adipose tissue plays a crucial role not only as an energy storage depot but also as an endocrine organ that secretes adipokines capable of modulating insulin sensitivity and inflammatory pathways [16]. Similarly, the liver contributes to metabolic imbalance through dysregulated gluconeogenesis, lipid accumulation, and the development of metabolic dysfunction–associated steatotic liver disease (MASLD) [17]. Skeletal muscle, the primary site of insulin-stimulated glucose uptake, becomes progressively resistant to insulin signaling, further contributing to hyperglycemia and metabolic inflexibility [18,19,20].

Chronic low-grade inflammation has also emerged as a central feature of T2DM pathogenesis [14]. Immune cell infiltration in adipose tissue, increased production of pro-inflammatory cytokines, and activation of intracellular pathways such as nuclear factor kappa B (NF-κB) and c-Jun N-terminal kinase (JNK) disrupt insulin signaling and promote metabolic dysfunction [21]. Importantly, inflammatory processes within the pancreas, including cytokine-mediated stress and immune cell infiltration in islets, further impair β-cell function and accelerate disease progression. Additionally, mitochondrial impairment and oxidative stress further exacerbate insulin resistance and β-cell dysfunction [4,16].

Given this complex multisystem network, increasing attention has been directed toward metabolic modulators that may influence these interconnected pathways [5,6,7,8]. Nutritional regulation and exercise have emerged as key strategies, capable of influencing systemic metabolism through both direct effects on tissues and indirect modulation of inter-organ signaling [22,23]. Simultaneously, targeting the gut microbiota has shown promise in shaping molecular pathways involved in energy balance, inflammation, and insulin signaling [24]. Among molecular modulators, vitamin D has gained particular interest due to its pleiotropic roles: beyond regulating calcium and bone metabolism, it influences immune function, mitochondrial activity, and inter-organ crosstalk via the vitamin D receptor (VDR), widely expressed in pancreatic β-cells, adipocytes, hepatocytes, and skeletal muscle. Notably, vitamin D may also modulate pancreatic inflammation, thereby contributing to the preservation of β-cell function and insulin secretion. Through these mechanisms, vitamin D may also contribute to the regulation of glucose homeostasis and glycemic control by modulating insulin secretion, insulin sensitivity, and inflammatory pathways associated with metabolic dysfunction [25,26]. Together, these approaches highlight a multifaceted framework for metabolic modulation, integrating nutrition, physical activity, microbiota, and molecular signaling.

Therefore, the aim of this review is to examine T2DM from a multisystem perspective, focusing on the molecular mechanisms underlying organ crosstalk in metabolic regulation. In addition, this review explores emerging metabolic modulators and its potential role in influencing key pathways involved in insulin resistance, adipose tissue dysfunction, mitochondrial metabolism, and chronic inflammation.

## 2. Pathophysiology of Type 2 Diabetes Mellitus as a Multisystem Disease

T2DM is one of the most prevalent chronic metabolic disorders worldwide and represents more than 90% of all diabetes cases [1,2]. It is characterized by persistent hyperglycemia resulting from a combination of peripheral insulin resistance and an inadequate compensatory insulin secretory response from pancreatic β-cells [4,16,19]. These defects disrupt glucose homeostasis and progressively impair metabolic regulation across multiple organs and tissues [10,22].

Under physiological conditions, insulin regulates glucose metabolism by promoting glucose uptake in peripheral tissues such as skeletal muscle and adipose tissue while suppressing hepatic glucose production [27,28]. In T2DM, however, insulin-responsive tissues become less sensitive to insulin signaling, a condition known as insulin resistance [3,12]. As a compensatory response, pancreatic β-cells initially increase insulin secretion to maintain normoglycemia [4,16]. Over time, chronic metabolic stress, glucotoxicity, lipotoxicity, and inflammation impair β-cell function, ultimately leading to insufficient insulin production and persistent hyperglycemia [29,30].

The pathogenesis of T2DM involves complex interactions between genetic predisposition, environmental influences, and lifestyle-related factors [31,32]. Obesity, particularly visceral adiposity, sedentary behavior, high-calorie diets, and aging are major drivers of the global increase in T2DM incidence [33,34,35]. Excess adipose tissue contributes to metabolic dysfunction through increased release of free fatty acids (FFAs) and dysregulated secretion of adipokines and inflammatory cytokines, which further promote insulin resistance and systemic metabolic imbalance [14,36,37].

Importantly, T2DM is now broadly understood as a multisystem disorder characterized by coordinated metabolic disturbances across multiple organs, including the pancreas, liver, skeletal muscle, adipose tissue, intestine, kidneys, and the central nervous system [38]. Alterations in this inter-organ communication network lead to disturbances in glucose and lipid metabolism, persistent low-grade inflammation, and a gradual worsening of metabolic function [14,38]. Emerging evidence also highlights the role of gut microbiota alterations, immune system activation, and mitochondrial dysfunction in the development and progression of the disease [15,24,38].

The pancreas plays a central role through insulin secretion, and its dysfunction contributes directly to impaired glycemic control. The liver regulates glucose production via gluconeogenesis and glycogenolysis, and its dysregulation leads to excessive hepatic glucose output. Skeletal muscle is the primary site of insulin-mediated glucose uptake, and insulin resistance in this tissue significantly reduces glucose disposal. Adipose tissue acts as an endocrine organ, releasing adipokines and inflammatory mediators that modulate systemic insulin sensitivity. The intestine contributes through nutrient absorption and incretin hormone secretion, Glucagon-like peptide-1 (GLP-1), which influences insulin secretion and appetite regulation. The kidneys participate in glucose homeostasis through glucose reabsorption, which is often increased in T2DM. The central nervous system regulates energy balance, appetite, and neuroendocrine signaling, thereby influencing systemic metabolism.

In addition to metabolic dysregulation, chronic hyperglycemia in T2DM leads to long-term complications affecting multiple organ systems, including cardiovascular disease, neuropathy, nephropathy, and retinopathy [19,39,40,41,42]. These complications significantly increase morbidity and mortality, emphasizing the need for a comprehensive understanding of the molecular and systemic mechanisms underlying the disease [43].

**Figure 1:**
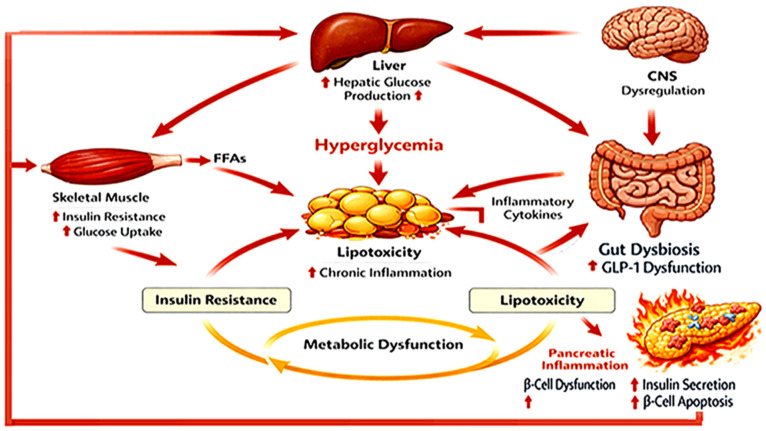
Multisystem Pathophysiology of T2DM. Source: Own elaboration.

Given the complex and multisystem nature of T2DM, current research increasingly focuses on the interconnected metabolic pathways that regulate insulin signaling, inflammation, oxidative stress, and mitochondrial function. Understanding these mechanisms is essential for identifying novel therapeutic targets and developing integrated strategies to prevent and manage this global metabolic disorder. In this context, Table 1 summarizes the principal clinical characteristics, diagnostic criteria, metabolic alterations, and major risk factors associated with T2DM, providing a concise overview of the key clinical and epidemiological aspects of the disease. Complementarily, Figure 1 illustrates the multisystem pathophysiology of T2DM, highlighting the interactions among adipose tissue dysfunction, impaired insulin signaling, chronic inflammation, and metabolic dysregulation that collectively contribute to disease onset and progression.

## 3. Molecular Signaling Pathways in Type 2 Diabetes Mellitus

The development and progression of T2DM are closely linked to alterations in intracellular signaling pathways that regulate cellular energy balance, nutrient sensing, and metabolic homeostasis [51]. In healthy metabolic conditions, multiple signaling networks coordinate glucose uptake, lipid metabolism, mitochondrial function, and inflammatory responses across different tissues [12,13,14,15,37,52]. However, in T2DM these regulatory systems become disrupted, leading to impaired insulin signaling, metabolic inflexibility, and chronic inflammation [14,53].

The earliest events in the pathophysiology of T2DM involve pancreatic inflammation, which plays a critical role in the disruption of β-cell function. Inflammatory processes within pancreatic islets, including immune cell infiltration and increased production of pro-inflammatory cytokines such as Tumor necrosis factor alpha (TNF-α) and Interleukin-1β (IL-1β), induce cellular stress and promote β-cell dysfunction and apoptosis [54,55]. This inflammatory microenvironment impairs insulin synthesis and secretion, representing an initial step in the progression toward metabolic dysregulation.

Among the most important pathways involved in metabolic regulation are the AMP-activated protein kinase (AMPK) pathway, the mechanistic target of rapamycin (mTOR) signaling cascade, and mitochondrial processes associated with oxidative stress [15,56,57,58]. These systems interact dynamically and influence key metabolic tissues including liver, skeletal muscle, adipose tissue, and pancreatic β-cells [16]. Dysregulation of these pathways contributes to insulin resistance, impaired glucose utilization, lipid accumulation, and progressive metabolic dysfunction [3,52,56].

Following pancreatic dysfunction, alterations in insulin signaling represent a central step in T2DM progression [59]. Components of the insulin signaling cascade, including insulin receptor substrate-1 (IRS-1), protein kinase B isoform 2 (AKT2), and glucose transporter type 4 (GLUT4), play central roles in mediating insulin-dependent glucose uptake in skeletal muscle and adipose tissue [60]. AKT2 is primarily expressed in skeletal muscle, adipose tissue, and liver, where it mediates insulin-stimulated glucose uptake and glycogen synthesis; its impairment leads to reduced glucose disposal and systemic insulin resistance. GLUT4 is predominantly located in skeletal muscle and adipocytes, where it facilitates insulin-dependent glucose transport into cells; its defective translocation contributes directly to hyperglycemia in T2DM. Dysregulation of these signaling elements contributes to impaired insulin action and reduced glucose utilization.

At the cytosolic level, metabolic alterations further compromise glucose utilization, particularly through impaired glycolysis. Insulin resistance reduces glucose entry into cells, limiting substrate availability for glycolytic pathways. In parallel, transcription factors involved in metabolic regulation, such as sterol regulatory element-binding protein-1c (SREBP-1c) and forkhead box protein O1 (FOXO1), contribute to metabolic imbalance. FOXO1 is mainly active in the liver, where it promotes gluconeogenic gene expression; its persistent activation in T2DM leads to excessive hepatic glucose production and fasting hyperglycemia, counteracting glycolytic processes and exacerbating systemic glucose dysregulation [61,62].

Inflammatory mediators also participate in metabolic dysfunction; for instance, TNF-α promotes chronic low-grade inflammation and interferes with insulin signaling pathways [61,62]. In addition, regulators of adipocyte differentiation and metabolic gene expression—including peroxisome proliferator-activated receptor gamma (PPARγ), CCAAT/enhancer-binding protein alpha (C/EBPα), fatty acid binding protein 4 (FABP4), bone morphogenetic protein 4 (BMP4), and Krüppel-like factor 5 (KLF5)—play essential roles in adipogenesis, lipid handling, and adipose tissue remodeling [63,64,65,66,67].

At the mitochondrial level, metabolic dysfunction is further exacerbated by oxidative stress and impaired energy production. Mitochondrial dysfunction reduces ATP synthesis and increases reactive oxygen species (ROS) production, which further damages cellular components and worsens insulin resistance [68]. These alterations are closely linked to dysregulation of AMPK and mTOR signaling pathways, which normally coordinate cellular energy sensing and nutrient availability [68].

Epigenetic and post-transcriptional regulatory mechanisms also contribute to metabolic dysfunction, particularly through fat mass and obesity-associated protein (FTO) and YTH N6-methyladenosine RNA binding protein 2 (YTHDF2), which influence ribonucleic acid (RNA) methylation dynamics and metabolic gene expression [69,70]. YTHDF2 is expressed in metabolically active tissues such as the liver and adipose tissue, where it regulates mRNA stability through m6A-dependent mechanisms; its dysregulation affects the expression of genes involved in lipid metabolism and insulin sensitivity. Additionally, platelet-derived growth factor receptor beta (PDGFRβ) participates in adipose tissue remodeling and stromal cell signaling, contributing to metabolic alterations associated with insulin resistance [71].

Together, these mechanisms form a complex molecular network linking nutrient sensing, gene regulation, inflammation, adipogenesis, and metabolic homeostasis in the development and progression of T2DM [13,14,35,37,69,70].

### 3.1. AMPK Signaling

AMPK is a highly conserved serine/threonine kinase that functions as a central regulator of cellular energy balance in health and disease [72,73]. AMPK is activated in response to increases in the intracellular AMP/ATP ratio, a signal indicating cellular energy depletion [74,75]. Once activated, AMPK promotes metabolic processes that generate ATP while simultaneously inhibiting energy-consuming anabolic pathways [75].

In metabolic tissues, AMPK activation enhances glucose uptake, stimulates fatty acid oxidation, and promotes mitochondrial biogenesis [76]. In skeletal muscle, AMPK facilitates the translocation of GLUT4 to the plasma membrane, thereby increasing glucose uptake independently of insulin signaling [77]. In the liver, AMPK suppresses gluconeogenesis and promotes lipid oxidation, contributing to improved metabolic balance [78]. Reduced AMPK activity has been observed in conditions associated with metabolic dysfunction, including obesity and T2DM [79]. Chronic nutrient excess, lipid accumulation, and inflammatory signaling may impair AMPK activation, thereby limiting the cell’s ability to maintain energy homeostasis [80]. This reduction in AMPK activity contributes to increased lipid storage, decreased mitochondrial function, and impaired glucose utilization [81].

Furthermore, AMPK interacts with several other signaling pathways involved in metabolic regulation, including mTOR signaling and pathways controlling oxidative stress responses [15,56,57,58]. Notably, AMPK acts as a key negative regulator of mTORC1 activity, linking cellular energy status with the control of anabolic processes. When energy availability is low, AMPK activation inhibits mTOR signaling, thereby preventing excessive energy expenditure and promoting metabolic adaptation. Through these interactions, AMPK serves as a key integrator of metabolic signals, coordinating cellular responses to energy availability and environmental stress [82]. Consequently, therapeutic strategies that enhance AMPK activation have been explored as potential interventions for improving insulin sensitivity and metabolic health in T2DM [83].

### 3.2. mTOR Signaling Pathway

mTOR is a serine/threonine kinase that plays a central role in regulating cell growth, protein synthesis, and nutrient sensing [84,85]. mTOR integrates signals derived from nutrients, growth factors, cellular energy status, and stress signals to coordinate anabolic and catabolic processes within the cell [84,85].

mTOR functions primarily through two distinct multiprotein complexes known as mTOR complex 1 (mTORC1) and mTOR complex 2 (mTORC2), each with different regulatory roles [86]. mTORC1 is particularly sensitive to nutrient availability and promotes protein synthesis, lipid biosynthesis, and cell growth when energy and nutrients are abundant [87]. In contrast, mTORC2 participates in the regulation of cytoskeletal organization and contributes to insulin signaling through activation of AKT [88,89].

In this context, mTOR signaling can be understood as a functional counterpart to AMPK, responding to conditions of nutrient abundance and energy sufficiency. While AMPK activation promotes catabolic pathways to restore energy balance, mTOR activation drives anabolic processes that support cell growth and biosynthesis. Under physiological conditions, a balance between AMPK and mTOR activity ensures appropriate metabolic adaptation to fluctuations in nutrient availability [90]. However, in metabolic disorders such as T2DM, chronic nutrient excess and elevated circulating glucose and lipid levels can lead to persistent activation of mTORC1 signaling [68].

This sustained activation of mTORC1, often coupled with reduced AMPK activity, disrupts the normal regulatory balance between energy sensing and nutrient signaling, thereby contributing to metabolic inflexibility. Excessive mTOR activity has also been associated with increased endoplasmic reticulum stress and inflammatory signaling, further contributing to metabolic dysfunction [91]. In pancreatic β-cells, dysregulated mTOR signaling may initially support compensatory insulin production but eventually contributes to cellular stress and β-cell failure [92].

The intricate interplay between AMPK and mTOR pathways is therefore crucial for maintaining metabolic homeostasis [84]. Thus, the coordinated regulation between AMPK-mediated energy sensing and mTOR-driven anabolic signaling represents a central axis in metabolic control, and its disruption constitutes a key molecular mechanism underlying insulin resistance and the progression of T2DM.

### 3.3. Oxidative Stress and Mitochondrial Dysfunction

Mitochondria play a fundamental role in cellular metabolism by generating ATP through oxidative phosphorylation and by regulating metabolic intermediates involved in glucose and lipid metabolism [93]. In addition to energy production, mitochondria are also a major source of ROS, which are produced as by-products of mitochondrial electron transport chain activity [94].

Under normal physiological conditions, ROS production is tightly controlled by antioxidant defense systems that maintain redox balance within the cell [95]. However, in metabolic disorders such as T2DM, excessive nutrient availability and mitochondrial overload can lead to increased ROS generation and oxidative stress [96].

Elevated levels of ROS can damage cellular components including proteins, lipids, and DNA, thereby disrupting normal cellular function [97]. In metabolic tissues such as skeletal muscle, liver, and adipose tissue, oxidative stress interferes with insulin signaling pathways and contributes to the development of insulin resistance [98]. Additionally, oxidative stress promotes inflammatory responses that further exacerbate metabolic dysfunction [99].

Mitochondrial dysfunction is also associated with impaired oxidative capacity, reduced ATP production, and accumulation of lipid intermediates within cells [15,100]. These alterations contribute to metabolic inflexibility and reduced capacity of tissues to efficiently utilize glucose and fatty acids as energy substrates [15,101].

In pancreatic β-cells, mitochondrial dysfunction and oxidative stress are particularly detrimental because these cells possess relatively low antioxidant defenses [4,16]. Excessive oxidative stress can impair insulin secretion and promote β-cell apoptosis, accelerating the progression of T2DM [102]. Given the central role of mitochondria in metabolic regulation, strategies aimed at improving mitochondrial function and reducing oxidative stress have attracted increasing attention as potential therapeutic approaches for metabolic diseases [103].

### 3.4. Molecular Regulators of Adipogenesis

Adipose tissue plays a central role in systemic metabolic regulation and represents one of the most dynamic endocrine organs involved in the pathophysiology of T2DM [104]. Beyond its function as an energy storage depot, adipose tissue regulates glucose and lipid metabolism through the secretion of adipokines, inflammatory mediators, and metabolic signaling molecules [105]. Consequently, alterations in adipocyte differentiation and adipose tissue expansion significantly contribute to the development of insulin resistance and metabolic dysfunction [106].

Adipogenesis is a highly regulated cellular process in which mesenchymal stem cells differentiate into mature adipocytes [107]. This process is controlled by a coordinated transcriptional cascade involving multiple transcription factors, signaling molecules, and epigenetic regulators that ensure proper adipocyte development and metabolic function [108].

Among the key transcriptional regulators, C/EBPα plays a fundamental role during the early stages of adipocyte differentiation [109,110]. This transcription factor activates genes involved in lipid metabolism and promotes the expression of PPARγ, which is widely recognized as the master regulator of adipogenesis [111]. Once activated, PPARγ drives the transcriptional program necessary for adipocyte maturation, including genes involved in lipid uptake, triglyceride storage, and insulin sensitivity, all of which are related to T2DM [53,63,64,112,113].

Another important regulator is SREBP1, which promotes lipid biosynthesis and contributes to adipocyte differentiation by enhancing PPARγ expression [112,113,114]. Through this mechanism, SREBP1 links lipid metabolism to adipogenic signaling pathways and facilitates lipid accumulation within developing adipocytes [115]. In addition to transcription factors, several metabolic proteins participate in adipocyte differentiation and metabolic regulation. FABP4, a cytoplasmic lipid chaperone highly expressed in adipocytes, modulates intracellular fatty acid trafficking and influences PPARγ activity [116]. Elevated FABP4 levels have been associated with obesity, chronic inflammation, and insulin resistance, suggesting that this protein may contribute to the metabolic alterations observed in T2DM [117].

Growth factors also play an important role in adipogenic commitment. BMP4 regulates the differentiation of precursor cells into adipocytes by promoting adipogenic lineage commitment and suppressing alternative stem cell [118]. BMP4 signaling contributes to adipocyte development partly through the induction of PPARγ expression and the downregulation of PDGFRβ, thereby facilitating adipogenic differentiation [119,120].

Recent advances in molecular biology have also highlighted the importance of epigenetic and post-transcriptional regulators in adipogenesis. For example, the FTO functions as an RNA demethylase that influences the stability and translation of messenger RNAs involved in adipocyte differentiation [121]. Through its demethylase activity, FTO regulates mRNA modifications such as m6A, thereby affecting early adipogenic processes and lipid metabolism [122,123].

Another RNA-binding protein involved in this regulatory network is YTHDF2, which modulates mRNA stability by targeting m6A-modified transcripts for degradation [124]. By regulating the stability of genes involved in cell cycle progression and differentiation, YTHDF2 acts as a negative regulator of adipogenesis, highlighting the importance of post-transcriptional mechanisms in adipocyte development [125].

Furthermore, nutrient-sensing pathways interact closely with transcriptional regulators during adipocyte differentiation. The mTOR pathway plays a critical role in coordinating cellular growth and metabolic signaling during adipogenesis [126]. Activation of mTOR signaling promotes lipid synthesis, energy metabolism, and insulin signaling, thereby supporting adipocyte maturation and metabolic activity [127,128].

Another transcription factor involved in early adipocyte differentiation is KLF5 and KLF9, which acts synergistically with other transcriptional regulators such as C/EBP proteins and PPARγ [129,130]. KLF5 participates in complex regulatory networks influenced by growth factors, circadian rhythms, and metabolic signaling pathways that collectively determine adipocyte fate and function [129].

Taken together, these transcriptional, metabolic, and epigenetic regulators form an intricate molecular network that governs adipocyte differentiation and adipose tissue expansion [131]. Dysregulation of these pathways can lead to abnormal adipose tissue remodeling, ectopic lipid accumulation, and chronic inflammation, all of which contribute to the development of insulin resistance and metabolic disorders such as T2DM [132].

**Figure 2:**
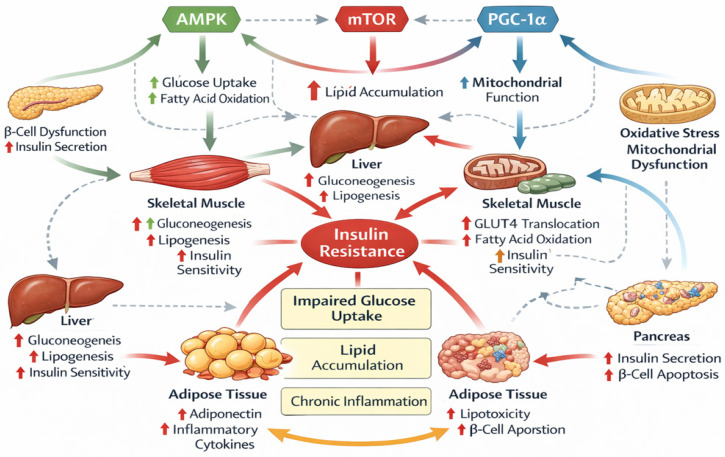
Molecular Signaling Pathways in T2DM. Source: Own elaboration.

Understanding the molecular mechanisms controlling adipogenesis is therefore essential for elucidating the role of adipose tissue in systemic metabolic regulation and for identifying potential therapeutic targets aimed at improving metabolic health. In this context, Table 2 summarizes key molecular regulators involved in adipogenesis, insulin signaling, energy sensing, epigenetic regulation, and inflammatory pathways associated with metabolic dysfunction. Complementarily, Figure 2 illustrates the major molecular signaling pathways implicated in the pathophysiology of T2DM, highlighting the complex interactions between metabolic, inflammatory, and transcriptional networks that contribute to disease progression.

## 4. Organ Crosstalk in Metabolic Regulation

Metabolic homeostasis is not regulated by isolated organs but rather by an integrated network of tissues that continuously exchange signals to coordinate energy balance, nutrient utilization, and metabolic adaptation [141]. The liver, adipose tissue, skeletal muscle, and gastrointestinal tract communicate through endocrine mediators, circulating metabolites, cytokines, and lipid-derived molecules that collectively regulate systemic metabolism [142]. Under physiological conditions, this inter-organ communication maintains glucose and lipid homeostasis by synchronizing nutrient storage, oxidation, and mobilization [52].

However, in metabolic disorders such as obesity and T2DM, these communication networks become disrupted [142]. Altered signaling between metabolic organs contributes to insulin resistance, chronic low-grade inflammation, and dysregulation of lipid and glucose metabolism [143]. Increasing evidence suggests that metabolic diseases arise not only from dysfunction within individual tissues but also from impaired coordination among metabolic organs [144]. Consequently, the concept of organ crosstalk has become an essential framework for understanding the systemic nature of metabolic dysregulation and identifying new targets for therapeutic intervention [10].

### 4.1. Adipose Tissue–Liver Axis

The adipose tissue–liver axis represents a fundamental pathway in the regulation of systemic metabolic homeostasis. Adipose tissue functions not only as a storage site for excess energy in the form of triglycerides but also as an active endocrine organ that secretes numerous bioactive molecules known as adipokines [145]. These include hormones and cytokines such as leptin, adiponectin, resistin, tumor necrosis factor alpha (TNF-α), and interleukin-6 (IL-6), which influence multiple metabolic processes including appetite regulation, lipid metabolism, and insulin sensitivity [12,54,55,146,147].

In the context of obesity and metabolic dysfunction, adipose tissue undergoes significant structural and functional alterations characterized by adipocyte hypertrophy, macrophage infiltration, and chronic inflammation [148]. These changes promote enhanced lipolysis and the excessive release of FFAs into the bloodstream [149]. Elevated circulating FFAs are transported to the liver, where they contribute to triglyceride accumulation and promote the development of hepatic steatosis [150]. This process is a central feature of MASLD, which frequently coexists with insulin resistance and T2DM [149,151].

In addition to lipid flux, dysregulated adipokine secretion plays a crucial role in modulating hepatic metabolism [152]. Reduced levels of adiponectin impair fatty acid oxidation and mitochondrial function in hepatocytes, while increased levels of pro-inflammatory cytokines activate stress-related signaling pathways that interfere with insulin receptor signaling [153]. These molecular disturbances lead to increased hepatic gluconeogenesis, reduced glycogen synthesis, and impaired insulin-mediated suppression of glucose production [52,154]. Consequently, the disruption of adipose tissue–liver communication contributes to systemic metabolic imbalance and the progression of insulin resistance [142].

### 4.2. Muscle–Liver Interaction

Skeletal muscle is the primary site of insulin-stimulated glucose uptake and therefore plays a pivotal role in maintaining systemic glucose homeostasis [155,156]. Through its capacity to utilize large quantities of glucose during both resting and active states, skeletal muscle strongly influences circulating glucose levels and overall metabolic balance [155,156]. Alterations in muscle metabolism can therefore have profound consequences for hepatic metabolic regulation and contribute to the pathogenesis of insulin resistance [157].

Communication between skeletal muscle and the liver occurs through multiple mechanisms, including the release of muscle-derived signaling molecules known as myokines [158]. These peptides and cytokines are secreted by muscle fibers in response to metabolic stress, contraction, or changes in nutrient availability [159]. Myokines such as irisin, IL-6, and fibroblast growth factor 21 (FGF21) have been shown to regulate hepatic glucose production, lipid metabolism, and inflammatory pathways [160,161,162,163]. Through these endocrine signals, skeletal muscle exerts a systemic influence that extends beyond its role in mechanical activity [158].

Metabolic intermediates generated during skeletal muscle metabolism also participate in muscle–liver communication [158]. For instance, altered glucose utilization in insulin-resistant muscle leads to elevated circulating glucose levels, which stimulate hepatic gluconeogenesis and contribute to hyperglycemia [10]. Similarly, changes in muscle lipid oxidation can increase circulating lipid intermediates that influence hepatic lipid handling and promote triglyceride accumulation in the liver [164].

Physical activity provides a clear example of the physiological relevance of this axis. Exercise stimulates glucose uptake in skeletal muscle independently of insulin and induces the release of beneficial myokines that improve hepatic insulin sensitivity and metabolic flexibility [165]. These adaptations highlight the dynamic interaction between skeletal muscle and liver metabolism and emphasize the importance of muscle function in the regulation of whole-body metabolic health [166,167].

### 4.3. Gut–Metabolism Axis

The gastrointestinal tract and its associated microbiota have emerged as key regulators of host metabolism [11,24]. The gut microbiome is composed of a complex community of microorganisms that interact with dietary components and host tissues to influence nutrient absorption, immune responses, and metabolic signaling pathways [168]. Growing evidence indicates that alterations in gut microbial composition, commonly referred to as dysbiosis, are associated with metabolic disorders including obesity, insulin resistance, and T2DM [11,24].

One of the primary mechanisms through which gut microbiota influence host metabolism is the production of short-chain fatty acids (SCFAs) such as acetate, propionate, and butyrate [169]. These metabolites are generated through the fermentation of dietary fibers and act as signaling molecules that regulate energy metabolism, intestinal barrier integrity, and inflammatory responses [170]. SCFAs can activate specific G-protein-coupled receptors and influence pathways involved in hepatic gluconeogenesis, lipid oxidation, and glucose homeostasis [171].

Gut microbiota also participate in the regulation of bile acid metabolism. Microbial enzymes modify primary bile acids synthesized in the liver, producing secondary bile acids that function as signaling molecules capable of activating metabolic receptors such as the farnesoid X receptor (FXR) and Takeda G-protein receptor 5 (TGR5) [172,173]. Activation of these receptors influences glucose metabolism, lipid regulation, and energy expenditure, thereby establishing an important link between intestinal microbial activity and systemic metabolic control [13].

In addition to metabolic signaling, the gut microbiome can modulate systemic inflammation through effects on intestinal barrier function. Increased intestinal permeability may allow the translocation of bacterial components such as lipopolysaccharides (LPS) into circulation, a phenomenon often referred to as metabolic endotoxemia [174]. This process activates inflammatory pathways that interfere with insulin signaling in peripheral tissues and contributes to the progression of metabolic disease [175]. Together, these mechanisms highlight the importance of the gut–metabolism axis as a central component of metabolic regulation and a promising target for therapeutic strategies aimed at improving metabolic health [11,24].

**Figure 3:**
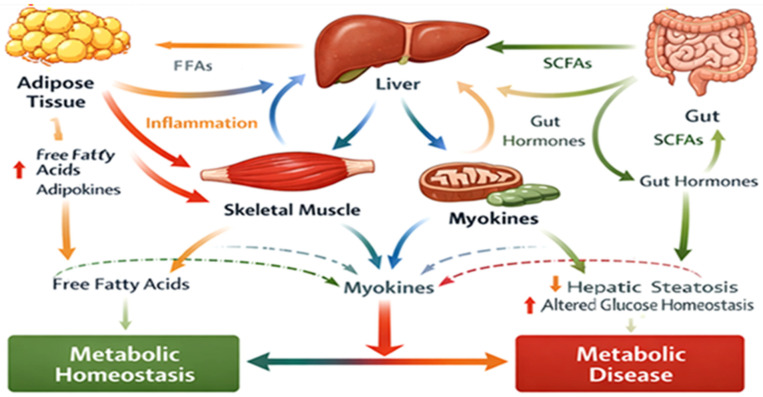
Organ Crosstalk in Metabolic Regulation. Source: Own elaboration.

To illustrate the systemic interactions involved in metabolic regulation, Figure 3 summarizes the key mechanisms of organ crosstalk associated with metabolic homeostasis. The diagram highlights the dynamic communication between the gut microbiota, liver, adipose tissue, skeletal muscle, and pancreas through metabolic signaling molecules, inflammatory mediators, and microbial metabolites. These interactions involve pathways related to SCFAs, bile acid signaling via FXR and TGR5 receptors, and inflammatory mediators such as LPS, which collectively influence glucose metabolism, lipid regulation, and insulin sensitivity. This integrative perspective emphasizes the importance of the gut–metabolism axis in the pathophysiology of metabolic disorders such as T2DM.

## 5. Lifestyle and Therapeutic Modulation of Molecular Signaling and Metabolic Crosstalk

Lifestyle factors represent some of the most powerful determinants of metabolic health and play a central role in regulating molecular signaling pathways involved in energy metabolism, inflammation, and inter-organ communication [176]. Environmental influences such as physical activity, nutritional patterns, micronutrient status, sleep quality, and circadian rhythms can profoundly affect the physiological mechanisms that maintain metabolic homeostasis [177,178,179]. In contrast, sedentary behavior and poor dietary habits contribute to the development of metabolic disorders including obesity, insulin resistance, and T2DM [180,181].

From a mechanistic perspective, lifestyle factors influence key molecular pathways that regulate cellular energy sensing, mitochondrial function, oxidative stress responses, and inflammatory signaling [176]. These pathways include energy sensors such as AMPK, nutrient-sensitive regulators like the mTOR, and transcriptional regulators involved in mitochondrial biogenesis and lipid metabolism [75,76,77,78,84,87,88,89,90]. Through the modulation of these signaling networks, lifestyle interventions can influence metabolic processes across multiple tissues including skeletal muscle, adipose tissue, liver, and the gastrointestinal tract [182,183,184].

Importantly, the effects of lifestyle factors extend beyond individual organs and involve coordinated interactions between metabolic tissues. Exercise, dietary composition, and micronutrient availability influence endocrine mediators such as myokines, adipokines, and hepatokines, which act as communication signals between organs [185,186]. These signals regulate processes including glucose uptake, lipid oxidation, inflammatory responses, and energy expenditure. Consequently, lifestyle behaviors play a crucial role in shaping the complex network of metabolic crosstalk that maintains systemic metabolic balance [177,178,179].

Growing evidence indicates that lifestyle interventions can restore metabolic flexibility, improve insulin sensitivity, and reduce chronic low-grade inflammation [187]. These beneficial effects occur through the simultaneous regulation of intracellular signaling pathways and inter-organ communication networks [188]. Understanding how lifestyle factors influence these molecular and physiological mechanisms is therefore essential for developing effective strategies to prevent and manage metabolic diseases [188].

### 5.1. Exercise-Induced Molecular and Metabolic Adaptations

Physical exercise is widely recognized as one of the most effective non-pharmacological strategies for improving metabolic health [189]. Regular physical activity induces a broad spectrum of physiological adaptations that influence glucose metabolism, lipid utilization, mitochondrial function, and inflammatory signaling [190,191]. These adaptations occur through the activation of multiple intracellular pathways that respond to the increased energetic demand associated with muscle contraction [192].

One of the most important molecular regulators activated during exercise is AMPK, a key cellular energy sensor that responds to changes in the AMP-to-ATP ratio [193]. During periods of energetic stress such as muscular contraction, AMPK activation promotes metabolic processes that restore cellular energy balance [194]. These include increased glucose uptake, enhanced fatty acid oxidation, and stimulation of mitochondrial biogenesis. Activation of AMPK also promotes the translocation of GLUT4 to the cell membrane, facilitating insulin-independent glucose uptake in skeletal muscle [195].

Exercise also modulates additional signaling pathways involved in metabolic adaptation. The mTOR pathway regulates protein synthesis, cellular growth, and nutrient sensing [196]. While AMPK primarily promotes catabolic pathways that generate energy, mTOR supports anabolic processes involved in tissue remodeling and muscle adaptation [196,197]. The dynamic balance between these pathways allows skeletal muscle to adapt to repeated exercise stimuli while maintaining metabolic efficiency.

Another critical regulator of exercise-induced adaptation is peroxisome proliferator-activated receptor gamma coactivator 1-alpha (PGC-1α), a transcriptional coactivator that promotes mitochondrial biogenesis and oxidative metabolism [198]. Exercise stimulates PGC-1α expression in skeletal muscle, leading to increased mitochondrial content and improved oxidative capacity [199]. Enhanced mitochondrial function contributes to greater metabolic flexibility, allowing cells to efficiently switch between carbohydrate and lipid substrates depending on energy demands [188].

Beyond intracellular signaling mechanisms, exercise also influences systemic metabolic regulation through the release of myokines. These muscle-derived signaling molecules act as endocrine mediators that communicate with other metabolic organs. Myokines such as irisin, IL-6, and FGF21 have been shown to influence lipid metabolism, adipose tissue function, and hepatic glucose production [200].

### 5.2. Nutritional Regulation of Metabolic Signaling

Dietary patterns play a fundamental role in shaping metabolic regulation by influencing nutrient availability, hormonal signaling, and the activity of metabolic pathways [201]. Macronutrient composition, caloric intake, and nutrient timing can all affect molecular signaling networks involved in glucose and lipid metabolism [202]. Nutritional factors interact with metabolic pathways such as AMPK, mTOR, and insulin signaling, thereby influencing cellular energy balance and metabolic adaptation [203].

For instance, caloric excess and high consumption of refined carbohydrates and saturated fats can disrupt metabolic homeostasis by promoting insulin resistance and chronic inflammation [204]. Excessive nutrient intake activates anabolic signaling pathways, particularly mTOR signaling, which may contribute to metabolic dysregulation when chronically stimulated [205]. At the same time, sustained nutrient overload may impair mitochondrial function and promote oxidative stress, further contributing to metabolic dysfunction [206].

Conversely, dietary strategies such as caloric restriction, intermittent fasting, and increased consumption of nutrient-dense foods can activate metabolic pathways associated with improved metabolic health [207]. These nutritional interventions often stimulate AMPK signaling and enhance mitochondrial function, thereby improving cellular energy metabolism and promoting metabolic flexibility [203].

Micronutrients also play an important role in metabolic regulation. Vitamins and minerals serve as cofactors for numerous enzymatic reactions involved in energy metabolism, antioxidant defense, and mitochondrial function [208]. Vitamin D, for example, has been associated with improved insulin sensitivity and modulation of inflammatory pathways, while other micronutrients such as magnesium and zinc participate in glucose metabolism and insulin signaling in diabetes [25,209]

In particular, vitamin D has been shown to influence pancreatic β-cell function, insulin secretion, and immune modulation through the VDR, which is widely expressed in metabolically active tissues [25]. However, despite these mechanistic insights, clinical evidence remains inconsistent. While observational studies frequently report an association between low vitamin D levels and increased risk of T2DM, randomized controlled trials have yielded mixed or inconclusive results regarding its effectiveness in improving glycemic control or preventing disease onset [25]. These discrepancies may be explained by differences in study design, baseline vitamin D status, dosage, duration of supplementation, and population heterogeneity [25].

Similarly, magnesium plays a key role in insulin receptor activity and glucose transport, particularly in skeletal muscle, and its deficiency has been associated with insulin resistance and increased risk of T2DM [25]. Zinc is essential for insulin synthesis, storage, and secretion in pancreatic β-cells, as well as for antioxidant defense mechanisms; however, supplementation studies have also shown variable outcomes depending on baseline nutritional status and metabolic conditions [209].

Beyond individual micronutrients, it is increasingly recognized that their effects are context-dependent and may interact with broader metabolic pathways, including AMPK and mTOR signaling, as well as mitochondrial function and oxidative stress [84]. This highlights the importance of considering micronutrient status as part of an integrated metabolic framework rather than as isolated therapeutic agents. Although micronutrients alone are unlikely to reverse metabolic disease, adequate micronutrient status supports the optimal functioning of metabolic pathways [210].

Dietary composition also influences the secretion of hormones and signaling molecules that participate in organ crosstalk [202]. Nutrient intake can regulate adipokine release from adipose tissue, hepatokine secretion from the liver, and gut-derived hormones involved in appetite regulation and glucose metabolism [211]. These signals contribute to the coordination of metabolic processes across multiple tissues and play an essential role in maintaining systemic metabolic balance [212].

### 5.3. Targeting Molecular Pathways in Metabolic Disease

One of the most promising areas of metabolic research involves the identification of molecular targets capable of restoring metabolic homeostasis. Among these targets, AMPK has received considerable attention due to its role as a central regulator of cellular energy balance [72,73,74,84]. Pharmacological activation of AMPK promotes glucose uptake, enhances fatty acid oxidation, and improves mitochondrial function, making it an attractive therapeutic target for metabolic disorders [213]. Several pharmacological agents used in the treatment of T2DM indirectly influence AMPK signaling, contributing to improved metabolic control [214].

Similarly, the mTOR signaling pathway represents an important regulator of cellular growth and nutrient sensing [85,87,90,205]. While excessive activation of mTOR has been associated with insulin resistance and metabolic dysregulation, controlled modulation of this pathway may provide therapeutic benefits. Understanding the balance between AMPK and mTOR signaling is therefore critical for designing interventions that support metabolic adaptation without disrupting essential cellular functions [15,56,57,58].

Mitochondrial dysfunction is another key feature of metabolic diseases and has emerged as a potential therapeutic target [15,100,208]. Strategies aimed at improving mitochondrial biogenesis, enhancing oxidative capacity, and reducing oxidative stress may contribute to improved metabolic efficiency. Interventions that stimulate transcriptional regulators such as PGC-1α may help restore mitochondrial function in metabolic tissues including skeletal muscle and liver [199].

In addition to these pathways, increasing attention has been directed toward regulators of adipogenesis and lipid metabolism. Transcription factors such as PPARs and SREBPs influence adipocyte differentiation and lipid storage, and their modulation may help regulate adipose tissue expansion and lipid distribution [133,136]. Targeting these pathways may reduce ectopic lipid accumulation and improve systemic insulin sensitivity.

### 5.4. Modulation of Inter-Organ Communication

The recognition that metabolic health depends on coordinated communication among organs has stimulated interest in therapies that modulate endocrine signaling between tissues. Skeletal muscle, adipose tissue, liver, and the gastrointestinal tract release numerous signaling molecules that influence metabolic regulation throughout the body. These include myokines, adipokines, hepatokines, and gut-derived metabolites, all of which participate in the regulation of glucose and lipid metabolism [10,141,144,176].

For example, myokines released during physical activity have been shown to improve metabolic function in distant tissues by promoting lipid oxidation, enhancing insulin sensitivity, and reducing inflammatory signaling [215,216]. Understanding how these molecules influence metabolic pathways may lead to the development of therapies that mimic or amplify exercise-induced signaling effects.

Similarly, adipokines produced by adipose tissue regulate appetite, insulin sensitivity, and lipid metabolism. Dysregulation of adipokine secretion in obesity contributes to chronic inflammation and metabolic imbalance [105,145,146,147]. Therapeutic strategies aimed at restoring healthy adipokine profiles may help reestablish metabolic communication between adipose tissue and other organs.

Hepatokines released by the liver also participate in metabolic regulation by influencing glucose metabolism, lipid handling, and systemic energy balance [217]. These molecules represent emerging targets for therapeutic interventions aimed at improving metabolic coordination between the liver and peripheral tissues [218].

The gut microbiome represents another important component of metabolic communication. Microbial metabolites such as SCFAs and modified bile acids act as signaling molecules that influence host metabolic pathways [219]. Interventions aimed at modifying the gut microbiota through dietary strategies, probiotics, or prebiotics may therefore provide novel approaches for regulating metabolic health [220].

### 5.5. Toward Precision and Integrative Metabolic Medicine

Future approaches to metabolic disease management are likely to incorporate concepts from precision medicine and systems biology [221,222]. Advances in genomic technologies, metabolomics, and microbiome research are enabling a more detailed understanding of the individual variability that influences metabolic responses to lifestyle and pharmacological interventions [223].

Personalized therapeutic strategies may consider factors such as genetic background, microbiome composition, metabolic phenotype, and environmental exposures [223]. This individualized approach could allow clinicians to tailor interventions that maximize metabolic benefits while minimizing adverse effects [221,222]. Moreover, integrating data from multiple biological systems may provide a more comprehensive understanding of metabolic regulation [221,222]. Systems biology approaches that analyze interactions between molecular signaling pathways, organ communication networks, and environmental influences may help identify new therapeutic targets and predictive biomarkers for metabolic disease progression [224].

Ultimately, future research should focus on developing integrative strategies that combine pharmacological treatments, lifestyle interventions, and personalized medicine approaches [225]. By addressing both the molecular mechanisms and systemic interactions underlying metabolic dysfunction, these strategies may offer more effective solutions for preventing and managing metabolic diseases [225].

**Figure 4:**
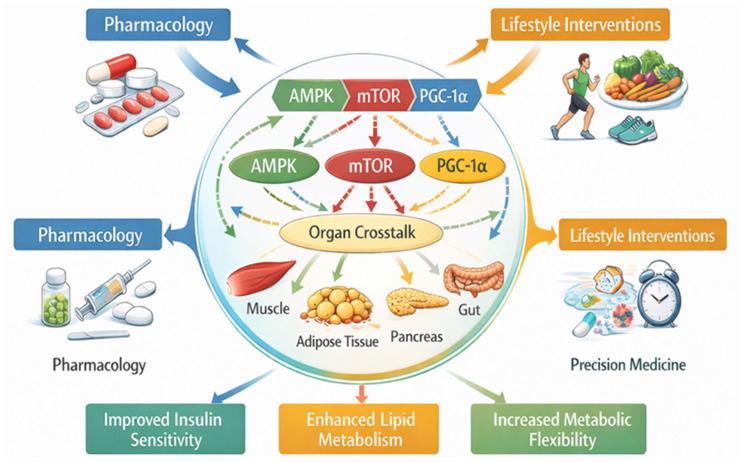
Integrated Therapeutic Strategies for Metabolic Diseases. Source: Own elaboration.

The pathophysiology of T2DM involves complex interactions between impaired insulin signaling, chronic low-grade inflammation, altered lipid metabolism, and dysregulated energy homeostasis [25]. Increasing evidence indicates that both lifestyle factors and molecular regulators contribute to the progression of metabolic dysfunction [226]. Interventions such as physical activity, dietary modifications, caloric restriction, and micronutrient status can influence key metabolic pathways involved in glucose transport, mitochondrial function, and inflammatory signaling [227,228]. Therefore, understanding how these strategies modulate specific molecular pathways is essential for developing effective preventive and therapeutic approaches. Table 3 summarizes the main lifestyle-based interventions and their associated molecular pathways and metabolic effects in T2DM. Complementarily, Figure 4 illustrates an integrated model of therapeutic strategies targeting multiple metabolic pathways involved in the management of metabolic diseases.

## 6. Clinical Implications and Future Research Directions

T2DM represents one of the most prevalent metabolic disorders worldwide and is characterized by chronic hyperglycemia resulting from insulin resistance and progressive pancreatic β-cell dysfunction. The pathophysiology of T2DM involves complex interactions among skeletal muscle, adipose tissue, liver, and the gastrointestinal tract, mediated through molecular signaling pathways and endocrine communication networks. Understanding these mechanisms provides important clinical insights for improving the prevention, diagnosis, and treatment of this disease.

From a therapeutic perspective, many current pharmacological strategies for T2DM target key molecular pathways involved in glucose metabolism and insulin signaling. Medications such as insulin sensitizers, incretin-based therapies, and sodium–glucose cotransporter 2 inhibitors act through mechanisms that improve insulin sensitivity, enhance glucose uptake, reduce hepatic glucose production, and promote glycosuria. These pharmacological approaches partially restore metabolic homeostasis by modulating pathways associated with cellular energy balance, inflammation, and lipid metabolism.

Nevertheless, pharmacological treatment alone is often insufficient to fully control disease progression. Lifestyle interventions remain a cornerstone of T2DM management and have demonstrated significant benefits in improving metabolic control. Regular physical activity enhances glucose uptake in skeletal muscle through insulin-independent mechanisms, largely mediated by the activation of AMPK and the translocation of GLUT4. In addition, exercise improves mitochondrial function, increases oxidative capacity, and reduces systemic inflammation, all of which contribute to improved insulin sensitivity.

Nutritional interventions also play a fundamental role in glycemic regulation. Dietary patterns that emphasize whole foods, fiber-rich carbohydrates, unsaturated fats, and adequate micronutrient intake can improve glycemic control and reduce metabolic risk. Caloric restriction and weight loss have been shown to significantly enhance insulin sensitivity and reduce hepatic lipid accumulation, which is closely associated with insulin resistance and metabolic dysfunction in T2DM.

Another important clinical aspect involves the role of inter-organ communication in glucose homeostasis. Skeletal muscle, adipose tissue, liver, and the gut secrete signaling molecules—including myokines, adipokines, hepatokines, and gut-derived metabolites—that influence insulin sensitivity and systemic metabolic regulation. Dysregulation of these signaling networks contributes to the development and progression of T2DM. Consequently, therapeutic strategies aimed at restoring balanced endocrine communication between metabolic tissues may represent promising targets for future interventions.

In recent years, advances in systems biology and multi-omics technologies have significantly expanded our understanding of the molecular complexity underlying T2DM. Approaches such as genomics, metabolomics, proteomics, and microbiome profiling are enabling the identification of novel biomarkers associated with disease susceptibility, metabolic phenotype, and treatment response. These technologies are also revealing substantial heterogeneity among individuals with T2DM, suggesting that personalized therapeutic strategies may be required to optimize clinical outcomes.

Future research should focus on elucidating the mechanisms that integrate lifestyle factors, molecular signaling pathways, and inter-organ communication in the development and progression of T2DM. Longitudinal studies investigating the combined effects of exercise, diet, and pharmacological therapies will be particularly important for identifying optimal strategies to restore metabolic homeostasis. Additionally, further investigation into the role of gut microbiota, mitochondrial function, and inflammatory signaling may provide new insights into disease mechanisms and therapeutic targets.

Ultimately, integrating molecular knowledge with lifestyle-based interventions and personalized medicine approaches may offer the most effective strategy for preventing and managing T2DM. A systems-level understanding of metabolic regulation will be essential for developing innovative treatments capable of addressing the multifactorial nature of this disease and improving long-term patient outcomes.

## 7. Conclusions

Metabolic diseases, including obesity and T2DM, arise from complex interactions between molecular, cellular, and systemic mechanisms. Dysregulation of key signaling pathways such as AMPK, mTOR, and mitochondrial networks impairs energy balance, glucose homeostasis, and lipid metabolism, contributing to insulin resistance and oxidative stress.

Inter-organ communication plays a central role in maintaining metabolic homeostasis. Crosstalk between adipose tissue, liver, skeletal muscle, and the gut integrates endocrine, metabolic, and inflammatory signals, influencing systemic energy regulation. Disruption of these networks exacerbates metabolic dysfunction.

Lifestyle factors, particularly physical activity and balanced nutrition, modulate these molecular and systemic pathways. Exercise enhances mitochondrial function, activates energy-sensing signaling, and improves insulin sensitivity, while diet and micronutrients influence metabolic signaling and gut microbiota composition. These interventions can restore metabolic flexibility and improve organ crosstalk.

In conclusion, understanding metabolic regulation requires integrating molecular signaling, organ communication, and environmental influences. Targeting these interconnected systems through combined pharmacological and lifestyle strategies offers the most promising approach for preventing and managing metabolic diseases.

## Figures and Tables

**Table 1:** Clinical characteristics, diagnostic criteria, and major risk factors associated with T2DM.

Category	Factor	Criteria	Reference
Diagnostic Criteria	Fasting plasma glucose	≥126 mg/dL after at least 8 h fasting.	[5,6,8,44]
	Oral glucose tolerance test	2 h plasma glucose ≥200 mg/dL after 75 g glucose load.	[5,6,8]
	HbA1c	≥6.5%, reflecting chronic hyperglycemia.	[5,6,8]
	Random plasma glucose	≥200 mg/dL in presence of classic hyperglycemia symptoms	[5,6,8]
	C-peptide	Relatively preserved.	[5,6,8]
	Ketoacidosis	Less frequent.	[5,6,8]
Major Risk Factors	Obesity	Excess adiposity, particularly visceral fat accumulation.	[44,45]
	Sedentary lifestyle	Low levels of physical activity associated with metabolic dysfunction.	[23,44]
	Family history	Genetic predisposition to T2DM among first-degree relatives.	[44,46]
	Age	Higher prevalence in adults and older populations.	[44,47]
	Sex	Higher prevalence in male sex	[44]
	Unhealthy diet	High intake of refined carbohydrates, fats, and processed foods.	[48]
	Hypertension	Frequently coexists with insulin resistance and metabolic syndrome.	[44,49]
	Dyslipidemia	Elevated LDL cholesterol and triglycerides.	[44,50]
	Cardiovascular disease	Increased risk of atherosclerosis and coronary heart disease.	[40]
	Anxiety	Psychological stress and anxiety disorders associated with poorer glycemic control and increased risk of T2DM.	[44]
	Depression	Depressive symptoms linked to metabolic dysregulation, reduced treatment adherence, and increased T2DM risk.	[44]

**Table 2:** Key molecular regulators involved in adipogenesis, insulin signaling, and metabolic dysfunction associated with T2DM.

Pathways	Gene/Protein	Function	References
Adipogenesis	PPARγ	Regulates adipocyte differentiation and insulin sensitivity	[133]
	FABP4	Modulates adipogenesis and lipid trafficking	[134]
	BMP4	Regulates commitment of precursor cells to the adipogenic lineage.	[135]
Epigenetic regulation	*C/EBPα*	Induces PPARγ expression, crucial for early adipocyte differentiation.	[136]
	FTO	Regulates RNA demethylation and metabolic gene expression.	[137]
	YTHDF2	Controls mRNA stability of N6-methyladenosine (m6A)-modified transcripts.	[138,139]
Insulin signaling	IRS-1	Key mediator of insulin signaling.	[60]
	AKT2	Central kinase in the insulin signaling pathway.	[60]
	GLUT4	Major insulin-dependent glucose transporter in muscle and adipose tissue.	[60,77]
Energy sensing	AMPK	Cellular energy sensor regulating glucose and lipid metabolism.	[21,51,56,72,73,74,75,76,77,78,79,80,81,82,83,84,90]
	mTOR	Central regulator of cell growth and nutrient sensing.	[57,80,85,86,87,88,89,90,126,127,128]
Metabolic regulation	SREBP-1c	Regulates genes involved in lipogenesis.	[61,140]
	FOXO1	Regulates gluconeogenesis and glucose metabolism.	[62]
Inflammation	TNF-α	Pro-inflammatory cytokine associated with insulin resistance.	[54,55]

**Table 3:** Lifestyle-based interventions for T2DM.

Intervention	Molecular Pathways Affected	Metabolic Effects	References
Physical exercise	AMPK ↑ PGC-1α ↑ GLUT4 ↑ translocation	Increased glucose uptake, improved mitochondrial function, enhanced insulin sensitivity	[229,230]
Caloric restriction	AMPK activation, mTOR inhibition	Improved metabolic flexibility and reduced adiposity	[231,232]
High-fiber diet	SCFA production, gut microbiota modulation	Improved glucose homeostasis and reduced inflammation	[233,234]
Vitamin D status	VDR signaling, immune modulation	Improved insulin sensitivity and inflammatory regulation	[25]
Intermittent fasting	AMPK activation, autophagy stimulation	Improved metabolic efficiency and reduced oxidative stress	[235]

## Data Availability

The data related to this study are available in this article.

## Abbreviations

The following abbreviations are used in this manuscript:

AKT2	Serine/threonine kinase 2.
AMPK	AMP-activated protein kinase.
ATP	Adenosine triphosphate.
BMP4	Bone morphogenetic protein 4.
C/EBPα	CCAAT/enhancer-binding protein alpha.
FABP4	Fatty acid-binding protein 4
FGF21	Fibroblast growth factor 21.
FFAs	Free fatty acids.
FOXO1	Forkhead box O1.
FXR	Farnesoid X receptor.
GLP-1	Glucagon-like peptide-1
GLUT4	Glucose transporter type 4.
HbA1c	Glycated hemoglobin.
IL-1β	Interleukin-1β
IL-6	Interleukin-6.
IRS-1	Insulin receptor substrate 1.
JNK	c-Jun N-terminal kinase.
KLF5	Krüppel-like factor 5.
LPS	Lipopolysaccharide.
MASLD	Metabolic dysfunction-associated steatotic liver disease.
m6A	N6-methyladenosine.
mRNA	Messenger ribonucleic acid.
mTOR	Mechanistic target of rapamycin.
mTORC1	mTOR complex 1.
mTORC2	mTOR complex 2.
NF-κB	Nuclear factor kappa B.
PDGFRβ	Platelet-derived growth factor receptor beta.
PPARγ	Peroxisome proliferator-activated receptor gamma.
RNA	Ribonucleic acid.
ROS	Reactive Oxygen Species.
SCFAs	Short-chain fatty acids.
SREBP1	Sterol regulatory element-binding protein 1.
T2DM	Type 2 diabetes mellitus.
TGR5	Takeda G-protein receptor 5.
TNF-α	Tumor necrosis factor alpha.
VDR	Vitamin D receptor.
YTHDF2	YTH N6-methyladenosine RNA binding protein 2.

## References

[1] Chen L., Magliano D.J., Zimmet P.Z. (2011). The worldwide epidemiology of type 2 diabetes mellitus--present and future perspectives. Nat. Rev. Endocrinol..

[2] Genitsaridi I., Salpea P., Salim A., Sajjadi S.F., Tomic D., James S., Thirunavukkarasu S., Issaka A., Chen L., Basit A. (2026). 11th edition of the IDF Diabetes Atlas: Global, regional, and national diabetes prevalence estimates for 2024 and projections for 2050. Lancet Diabetes Endocrinol..

[3] Accili D., Deng Z., Liu Q. (2025). Insulin resistance in type 2 diabetes mellitus. Nat. Rev. Endocrinol..

[4] Gao Y., Chen Q., Wu Z., Yuan L. (2025). Regulation of pancreatic β cells by exosomes from different sources. Diabetes Res. Clin. Pract..

[5] Harreiter J., Roden M. (2023). Diabetes Mellitus: Definition, Classification, Diagnosis, Screening, and Prevention (Update 2023). Wien. Klin. Wochenschr..

[6] Schleicher E., Gerdes C., Petersmann A., Müller-Wieland D., Müller U.A., Freckmann G., Heinemann L., Nauck M., Landgraf R. (2022). Definition, Classification, and Diagnosis of Diabetes Mellitus. Exp. Clin. Endocrinol. Diabetes.

[7] Antar S.A., Ashour N.A., Sharaky M., Khattab M., Ashour N.A., Zaid R.T., Roh E.J., Elkamhawy A., Al-Karmalawy A.A. (2023). Diabetes Mellitus: Classification, Mediators, and Complications; A Gate to Identify Potential Targets for the Development of New Effective Treatments. Biomed. Pharmacother..

[8] American Diabetes Association Professional Practice Committee (2024). 2. Diagnosis and Classification of Diabetes: Standards of Care in Diabetes—2024. Diabetes Care.

[9] Tian X., Wang L., Zhong L., Zhang K., Ge X., Luo Z., Zhai X., Liu S. (2025). The research progress and future directions in the pathophysiological mechanisms of type 2 diabetes mellitus from the perspective of precision medicine. Front. Med..

[10] Xourafa G., Korbmacher M., Roden M. (2024). Inter-organ crosstalk during development and progression of type 2 diabetes mellitus. Nat. Rev. Endocrinol..

[11] Yao W., Huo J., Ji J., Liu K., Tao P. (2024). Elucidating the role of gut microbiota metabolites in diabetes by employing network pharmacology. Mol. Med..

[12] Rehman K., Akash M.S.H., Liaqat A., Kamal S., Qadir M.I., Rasul A. (2017). Role of Interleukin-6 in Development of Insulin Resistance and Type 2 Diabetes Mellitus. Crit. Rev. Eukaryot. Gene Expr..

[13] Chávez-Talavera O., Tailleux A., Lefebvre P., Staels B. (2017). Bile Acid Control of Metabolism and Inflammation in Obesity, Type 2 Diabetes, Dyslipidemia, and Nonalcoholic Fatty Liver Disease. Gastroenterology.

[14] Guo W., Song Y., Sun Y., Du H., Cai Y., You Q., Fu H., Shao L. (2022). Systemic immune-inflammation index is associated with diabetic kidney disease in Type 2 diabetes mellitus patients: Evidence from NHANES 2011-2018. Front. Endocrinol..

[15] Pinti M.V., Fink G.K., Hathaway Q.A., Durr A.J., Kunovac A., Hollander J.M. (2019). Mitochondrial dysfunction in type 2 diabetes mellitus: An organ-based analysis. Am. J. Physiol. Endocrinol. Metab..

[16] Murakami T., Inagaki N., Kondoh H. (2022). Cellular Senescence in Diabetes Mellitus: Distinct Senotherapeutic Strategies for Adipose Tissue and Pancreatic β Cells. Front. Endocrinol..

[17] Ferdous S.E., Ferrell J.M. (2024). Pathophysiological Relationship between Type 2 Diabetes Mellitus and Metabolic Dysfunction-Associated Steatotic Liver Disease: Novel Therapeutic Approaches. Int. J. Mol. Sci..

[18] Lopez-Pedrosa J.M., Camprubi-Robles M., Guzman-Rolo G., Lopez-Gonzalez A., Garcia-Almeida J.M., Sanz-Paris A., Rueda R. (2024). The Vicious Cycle of Type 2 Diabetes Mellitus and Skeletal Muscle Atrophy: Clinical, Biochemical, and Nutritional Bases. Nutrients.

[19] Davies M.J., Aroda V.R., Collins B.S., Gabbay R.A., Green J., Maruthur N.M., Rosas S.E., Del Prato S., Mathieu C., Mingrone G. (2022). Management of hyperglycaemia in type 2 diabetes, 2022. A consensus report by the American Diabetes Association (ADA) and the European Association for the Study of Diabetes (EASD). Diabetologia.

[20] Hara K., Sakai Y., Tajiri Y., Nomura M. (2022). Beneficial effects of SGLT2 inhibitor on metabolic inflexibility and visceral fat amount in animal model of obese type 2 diabetes. Heliyon.

[21] Andreasen A.S., Kelly M., Berg R.M., Møller K., Pedersen B.K. (2011). Type 2 diabetes is associated with altered NF-κB DNA binding activity, JNK phosphorylation, and AMPK phosphorylation in skeletal muscle after LPS. PLoS ONE.

[22] Yang W., Jiang W., Guo S. (2023). Regulation of Macronutrients in Insulin Resistance and Glucose Homeostasis during Type 2 Diabetes Mellitus. Nutrients.

[23] Kanaley J.A., Colberg S.R., Corcoran M.H., Malin S.K., Rodriguez N.R., Crespo C.J., Kirwan J.P., Zierath J.R. (2022). Exercise/Physical Activity in Individuals with Type 2 Diabetes: A Consensus Statement from the American College of Sports Medicine. Med. Sci. Sports Exerc..

[24] Zhou Z., Sun B., Yu D., Zhu C. (2022). Gut Microbiota: An Important Player in Type 2 Diabetes Mellitus. Front. Cell. Infect. Microbiol..

[25] Fuentes-Barría H., Aguilera-Eguía R., Flores-Fernández C., Angarita-Davila L., Rojas-Gómez D., Alarcón-Rivera M., López-Soto O., Maureira-Sánchez J. (2025). Vitamin D and Type 2 Diabetes Mellitus: Molecular Mechanisms and Clinical Implications—A Narrative Review. Int. J. Mol. Sci..

[26] Zhang Y., Tan H., Tang J., Li J., Chong W., Hai Y., Feng Y., Lunsford L.D., Xu P., Jia D. (2020). Effects of Vitamin D Supplementation on Prevention of Type 2 Diabetes in Patients With Prediabetes: A Systematic Review and Meta-analysis. Diabetes Care.

[27] Raza G.S., Herzig K.H. (2022). Hepatic glucose production and storage as a potential strategy for type 2 diabetes treatment—The effect of catestatin—“just another new kid in town?”. Acta Physiol..

[28] Foretz M., Guigas B., Viollet B. (2019). Understanding the glucoregulatory mechanisms of metformin in type 2 diabetes mellitus. Nat. Rev. Endocrinol..

[29] Poitout V., Robertson R.P. (2008). Glucolipotoxicity: Fuel excess and beta-cell dysfunction. Endocr. Rev..

[30] Wysham C., Shubrook J. (2020). Beta-cell failure in type 2 diabetes: Mechanisms, markers, and clinical implications. Postgrad. Med..

[31] Veerabathiran R., P A., BK I., D R., RS A.H. (2023). Genetic predisposition of LEPR (rs1137101) gene polymorphism related to type 2 diabetes mellitus—A meta-analysis. Ann. Med..

[32] Xu Z., Lin R., Ji X., Huang C., Wang C., Yu Y., Bao Z. (2025). Physical frailty, genetic predisposition, and type 2 diabetes mellitus. Diabetes Metab..

[33] Khalafi M., Fatolahi S., Symonds M.E., Dinizadeh F., Rosenkranz S.K., Batrakoulis A. (2026). Comparative Efficacy of Exercise Type on Visceral Adipose Tissue in Patients with Prediabetes and Type 2 Diabetes Mellitus: A Systematic Review With Pairwise and Network Meta-Analyses. Obes. Rev..

[34] Forouhi N.G. (2023). Embracing complexity: Making sense of diet, nutrition, obesity and type 2 diabetes. Diabetologia.

[35] Ren X., Yang X., Jiang H., Han T., Sun C. (2021). The association of energy and macronutrient intake at dinner vs breakfast with the incidence of type 2 diabetes mellitus in a cohort study: The China Health and Nutrition Survey, 1997–2011. J. Diabetes.

[36] Blüher M. (2024). Understanding Adipose Tissue Dysfunction. J. Obes. Metab. Syndr..

[37] He F., Huang Y., Song Z., Zhou H.J., Zhang H., Perry R.J., Shulman G.I., Min W. (2021). Mitophagy-mediated adipose inflammation contributes to type 2 diabetes with hepatic insulin resistance. J. Exp. Med..

[38] Guo H., Pan L., Wu Q., Wang L., Huang Z., Wang J., Wang L., Fang X., Dong S., Zhu Y. (2025). Type 2 Diabetes and the Multifaceted Gut-X Axes. Nutrients.

[39] Liu L., Ke W., Li H., Li F., Fan G., Kuang J., Ma J., Zhang X., Ji B., Li S. (2024). Intense simplified strategy for newly diagnosed type 2 diabetes in patients with severe hyperglycaemia: Multicentre, open label, randomised trial. BMJ.

[40] Cao B., Guo Z., Li D.T., Zhao L.Y., Wang Z., Gao Y.B., Wang Y.X. (2025). The association between stress-induced hyperglycemia ratio and cardiovascular events as well as all-cause mortality in patients with chronic kidney disease and diabetic nephropathy. Cardiovasc. Diabetol..

[41] Grisold A., Callaghan B.C., Feldman E.L. (2017). Mediators of diabetic neuropathy: Is hyperglycemia the only culprit?. Curr. Opin. Endocrinol. Diabetes Obes..

[42] Kaur G., Harris N.R. (2023). Endothelial glycocalyx in retina, hyperglycemia, and diabetic retinopathy. Am. J. Physiol. Cell Physiol..

[43] Pasquel F.J., Lansang M.C., Dhatariya K., Umpierrez G.E. (2021). Management of diabetes and hyperglycaemia in the hospital. Lancet Diabetes Endocrinol..

[44] Hu S., Ji W., Zhang Y., Zhu W., Sun H., Sun Y. (2025). Risk factors for progression to type 2 diabetes in prediabetes: A systematic review and meta-analysis. BMC Public Health.

[45] Purnell J.Q. (2023). What Is Obesity? Definition as a Disease, With Implications for Care. Gastroenterol. Clin. N. Am..

[46] Chung J., Miller B.J. (2020). Meta-Analysis of Comorbid Diabetes and Family History of Diabetes in Non-Affective Psychosis. Schizophr. Res..

[47] Kautzky-Willer A., Leutner M., Harreiter J. (2023). Sex Differences in Type 2 Diabetes. Diabetologia.

[48] Ma X., Nan F., Liang H., Shu P., Fan X., Song X., Hou Y., Zhang D. (2022). Excessive Intake of Sugar: An Accomplice of Inflammation. Front. Immunol..

[49] Vargas-Uricoechea H., Cáceres-Acosta M.F. (2019). Blood Pressure Control and Impact on Cardiovascular Events in Patients with Type 2 Diabetes Mellitus: A Critical Analysis of the Literature. Clin. Investig. Arterioscler..

[50] Perego C., Da Dalt L., Pirillo A., Galli A., Catapano A.L., Norata G.D. (2019). Cholesterol Metabolism, Pancreatic β-Cell Function and Diabetes. Biochim. Biophys. Acta Mol. Basis Dis..

[51] Hunie Tesfa K., Desalegn Gebeyehu C., Tadele Ewunetie A., Gugsa E., Nigatu Zewdie A., Dessie G., Tezera Endale H. (2025). The role of AMPK signaling pathway in the pathogenesis of type 2 diabetes mellitus with its complications and related metabolic disorders. Metabol. Open.

[52] Petersen M.C., Shulman G.I. (2018). Mechanisms of Insulin Action and Insulin Resistance. Physiol. Rev..

[53] Gilardi F., Winkler C., Quignodon L., Diserens J.G., Toffoli B., Schiffrin M., Sardella C., Preitner F., Desvergne B. (2019). Systemic PPARγ deletion in mice provokes lipoatrophy, organomegaly, severe type 2 diabetes and metabolic inflexibility. Metabolism.

[54] Chen Y.L., Qiao Y.C., Xu Y., Ling W., Pan Y.H., Huang Y.C., Geng L.J., Zhao H.L., Zhang X.X. (2017). Serum TNF-α concentrations in type 2 diabetes mellitus patients and diabetic nephropathy patients: A systematic review and meta-analysis. Immunol. Lett..

[55] Swaroop J.J., Rajarajeswari D., Naidu J.N. (2012). Association of TNF-α with insulin resistance in type 2 diabetes mellitus. Indian J. Med. Res..

[56] Madhavi Y.V., Gaikwad N., Yerra V.G., Kalvala A.K., Nanduri S., Kumar A. (2019). Targeting AMPK in Diabetes and Diabetic Complications: Energy Homeostasis, Autophagy and Mitochondrial Health. Curr. Med. Chem..

[57] Yang L., Zhang Z., Wang D., Jiang Y., Liu Y. (2022). Targeting mTOR Signaling in Type 2 Diabetes Mellitus and Diabetes Complications. Curr. Drug Targets.

[58] Rovira-Llopis S., Bañuls C., Diaz-Morales N., Hernandez-Mijares A., Rocha M., Victor V.M. (2017). Mitochondrial dynamics in type 2 diabetes: Pathophysiological implications. Redox Biol..

[59] Su J., Luo Y., Hu S., Tang L., Ouyang S. (2023). Advances in Research on Type 2 Diabetes Mellitus Targets and Therapeutic Agents. Int. J. Mol. Sci..

[60] Suaifan G.A.R.Y., Alkhawaja B., Shehadeh M.B., Sharmaa M., Hor Kuan C., Okechukwu P.N. (2024). Glucosamine substituted sulfonylureas: IRS-PI3K-PKC-AKT-GLUT4 insulin signalling pathway intriguing agent. RSC Med. Chem..

[61] Adhikary P., Banerjee S., Dey B.K., Gargari P., Chatterjee S., Chakraborty D., Chowdhury S. (2024). Association of adipocyte size and SREBP-1c in visceral and subcutaneous adipose tissue in non-obese type 2 diabetes mellitus. Endocrine.

[62] Ge W., Zhao Y., Yang Y., Ding Z., Xu X., Weng D., Wang S., Cheng R., Zhang J. (2021). An insulin-independent mechanism for transcriptional regulation of Foxo1 in type 2 diabetic mice. J. Biol. Chem..

[63] Lavecchia A., Cerchia C. (2018). Selective PPARγ modulators for Type 2 diabetes treatment: How far have we come and what does the future hold?. Future Med. Chem..

[64] Zhu G., Zhang J., Yue L., Xiang G. (2022). Preventive effect of salicin ether against type-2 diabetes mellitus through targeting PPARγ-regulated gene expression. Acta Biochim. Pol..

[65] Furuhashi M., Sakuma I., Morimoto T., Higashiura Y., Sakai A., Matsumoto M., Sakuma M., Shimabukuro M., Nomiyama T., Arasaki O. (2020). Independent and Distinct Associations of FABP4 and FABP5 With Metabolic Parameters in Type 2 Diabetes Mellitus. Front. Endocrinol..

[66] Tang S., Zhang R., Yu W., Jiang F., Wang J., Chen M., Peng D., Yan J., Bao Y., Jia W. (2013). Association of genetic variants of BMP4 with type 2 diabetes mellitus and clinical traits in a Chinese Han population. Biomed Res. Int..

[67] Wang X.H., Yan C.Y., Liu J.R. (2019). Hyperinsulinemia-induced KLF5 mediates endothelial angiogenic dysfunction in diabetic endothelial cells. J. Mol. Histol..

[68] Wang H., Chen J., Bai G., Han W., Guo R., Cui N. (2022). mTOR Modulates the Endoplasmic Reticulum Stress-Induced CD4^+^ T Cell Apoptosis Mediated by ROS in Septic Immunosuppression. Mediat. Inflamm..

[69] Amine Ikhanjal M., Ali Elouarid M., Zouine C., El Alami H., Errafii K., Ghazal H., Alidrissi N., Bakkali F., Benmoussa A., Hamdi S. (2023). FTO gene variants (rs9939609, rs8050136 and rs17817449) and type 2 diabetes mellitus risk: A Meta-Analysis. Gene.

[70] Zheng J., Chen X., Wu L., Zhou Y., Wang Z., Li J., Liu Y., Peng G., Berggren P.O., Zheng X. (2021). Identification of MDM2, YTHDF2 and DDX21 as potential biomarkers and targets for treatment of type 2 diabetes. Biochem. Biophys. Res. Commun..

[71] Onogi Y., Wada T., Kamiya C., Inata K., Matsuzawa T., Inaba Y., Kimura K., Inoue H., Yamamoto S., Ishii Y. (2017). PDGFRβ Regulates Adipose Tissue Expansion and Glucose Metabolism via Vascular Remodeling in Diet-Induced Obesity. Diabetes.

[72] Wang B., Cheng K.K. (2018). Hypothalamic AMPK as a Mediator of Hormonal Regulation of Energy Balance. Int. J. Mol. Sci..

[73] López M. (2022). Hypothalamic AMPK as a possible target for energy balance-related diseases. Trends Pharmacol. Sci..

[74] Ke R., Xu Q., Li C., Luo L., Huang D. (2018). Mechanisms of AMPK in the maintenance of ATP balance during energy metabolism. Cell Biol. Int..

[75] Herzig S., Shaw R.J. (2018). AMPK: Guardian of metabolism and mitochondrial homeostasis. Nat. Rev. Mol. Cell Biol..

[76] Malik N., Ferreira B.I., Hollstein P.E., Curtis S.D., Trefts E., Weiser Novak S., Yu J., Gilson R., Hellberg K., Fang L. (2023). Induction of lysosomal and mitochondrial biogenesis by AMPK phosphorylation of FNIP1. Science.

[77] Zhang C., Jiang Y., Liu J., Jin M., Qin N., Chen Y., Niu W., Duan H. (2018). AMPK/AS160 mediates tiliroside derivatives-stimulated GLUT4 translocation in muscle cells. Drug Des. Dev. Ther..

[78] Johanns M., Hue L., Rider M.H. (2023). AMPK inhibits liver gluconeogenesis: Fact or fiction?. Biochem. J..

[79] Wang D., Yang L., Liu Y. (2022). Targeting AMPK Signaling in the Liver: Implications for Obesity and Type 2 Diabetes Mellitus. Curr. Drug Targets.

[80] Li H., Min Q., Ouyang C., Lee J., He C., Zou M.H., Xie Z. (2014). AMPK activation prevents excess nutrient-induced hepatic lipid accumulation by inhibiting mTORC1 signaling and endoplasmic reticulum stress response. Biochim. Biophys. Acta.

[81] Li X., Zhang X., Shen Z., Chen Z., Wang H., Zhang X. (2022). GnRH receptor mediates lipid storage in female adipocytes via AMPK pathway. Int. J. Med. Sci..

[82] Lee H., Zandkarimi F., Zhang Y., Meena J.K., Kim J., Zhuang L., Tyagi S., Ma L., Westbrook T.F., Steinberg G.R. (2020). Energy-stress-mediated AMPK activation inhibits ferroptosis. Nat. Cell Biol..

[83] Guo T., Yan W., Cui X., Liu N., Wei X., Sun Y., Fan K., Liu J., Zhu Y., Wang Z. (2023). Liraglutide attenuates type 2 diabetes mellitus-associated non-alcoholic fatty liver disease by activating AMPK/ACC signaling and inhibiting ferroptosis. Mol. Med..

[84] González A., Hall M.N., Lin S.C., Hardie D.G. (2020). AMPK and TOR: The Yin and Yang of Cellular Nutrient Sensing and Growth Control. Cell Metab..

[85] Liu G.Y., Sabatini D.M. (2020). mTOR at the nexus of nutrition, growth, ageing and disease. Nat. Rev. Mol. Cell Biol..

[86] Szwed A., Kim E., Jacinto E. (2021). Regulation and metabolic functions of mTORC1 and mTORC2. Physiol. Rev..

[87] Goul C., Peruzzo R., Zoncu R. (2023). The molecular basis of nutrient sensing and signalling by mTORC1 in metabolism regulation and disease. Nat. Rev. Mol. Cell Biol..

[88] Tsuji-Tamura K., Ogawa M. (2018). Dual inhibition of mTORC1 and mTORC2 perturbs cytoskeletal organization and impairs endothelial cell elongation. Biochem. Biophys. Res. Commun..

[89] Martinez Calejman C., Trefely S., Entwisle S.W., Luciano A., Jung S.M., Hsiao W., Torres A., Hung C.M., Li H., Snyder N.W. (2020). mTORC2-AKT signaling to ATP-citrate lyase drives brown adipogenesis and de novo lipogenesis. Nat. Commun..

[90] Sukumaran A., Choi K., Dasgupta B. (2020). Insight on Transcriptional Regulation of the Energy Sensing AMPK and Biosynthetic mTOR Pathway Genes. Front. Cell Dev. Biol..

[91] De Vita V., Melnik B.C. (2019). Activation of mechanistic target of rapamycin complex 1: The common link between rheumatoid arthritis and diabetes mellitus. Rheumatology.

[92] Wang J., Yang X., Zhang J. (2016). Bridges between mitochondrial oxidative stress, ER stress and mTOR signaling in pancreatic β cells. Cell. Signal..

[93] Garde A., Kenny I.W., Kelley L.C., Chi Q., Mutlu A.S., Wang M.C., Sherwood D.R. (2022). Localized glucose import, glycolytic processing, and mitochondria generate a focused ATP burst to power basement-membrane invasion. Dev. Cell.

[94] Palma F.R., Gantner B.N., Sakiyama M.J., Kayzuka C., Shukla S., Lacchini R., Cunniff B., Bonini M.G. (2024). ROS production by mitochondria: Function or dysfunction?. Oncogene.

[95] Court A.C., Vega-Letter A.M., Parra-Crisóstomo E., Velarde F., García C., Ortloff A., Vernal R., Pradenas C., Luz-Crawford P., Khoury M. (2024). Mitochondrial transfer balances cell redox, energy and metabolic homeostasis in the osteoarthritic chondrocyte preserving cartilage integrity. Theranostics.

[96] Rendra E., Riabov V., Mossel D.M., Sevastyanova T., Harmsen M.C., Kzhyshkowska J. (2019). Reactive oxygen species (ROS) in macrophage activation and function in diabetes. Immunobiology.

[97] Azzouz D., Palaniyar N. (2024). How Do ROS Induce NETosis? Oxidative DNA Damage, DNA Repair, and Chromatin Decondensation. Biomolecules.

[98] Russell-Guzmán J., Américo-Da Silva L., Cadagan C., Maturana M., Palomero J., Estrada M., Barrientos G., Buvinic S., Hidalgo C., Llanos P. (2024). Activation of the ROS/TXNIP/NLRP3 pathway disrupts insulin-dependent glucose uptake in skeletal muscle of insulin-resistant obese mice. Free Radic. Biol. Med..

[99] Lima J.E.B.F., Moreira N.C.S., Sakamoto-Hojo E.T. (2022). Mechanisms underlying the pathophysiology of type 2 diabetes: From risk factors to oxidative stress, metabolic dysfunction, and hyperglycemia. Mutat. Res. Genet. Toxicol. Environ. Mutagen..

[100] Lin S., Wu B., Hu X., Lu H. (2024). Sirtuin 4 (Sirt4) downregulation contributes to chondrocyte senescence and osteoarthritis via mediating mitochondrial dysfunction. Int. J. Biol. Sci..

[101] Wang X., Menezes C.J., Jia Y., Xiao Y., Venigalla S.S.K., Cai F., Hsieh M.H., Gu W., Du L., Sudderth J. (2024). Metabolic inflexibility promotes mitochondrial health during liver regeneration. Science.

[102] Shao L., Kong X., Lv S., Shu X., Ma X., Ai X., Yan D., Ying Y. (2024). FXR-regulated COX6A2 triggers mitochondrial apoptosis of pancreatic β-cell in type 2 diabetes. Cell Death Dis..

[103] Fromenty B., Roden M. (2023). Mitochondrial alterations in fatty liver diseases. J. Hepatol..

[104] Cheng L., Wang J., Dai H., Duan Y., An Y., Shi L., Lv Y., Li H., Wang C., Ma Q. (2021). Brown and beige adipose tissue: A novel therapeutic strategy for obesity and type 2 diabetes mellitus. Adipocyte.

[105] Francisco V., Pino J., Gonzalez-Gay M.A., Mera A., Lago F., Gómez R., Mobasheri A., Gualillo O. (2018). Adipokines and inflammation: Is it a question of weight?. Br. J. Pharmacol..

[106] Vishvanath L., Gupta R.K. (2019). Contribution of adipogenesis to healthy adipose tissue expansion in obesity. J. Clin. Investig..

[107] Hade M.D., Butsch B.L., Loreto Palacio P., Nguyen K.T., Shantaram D., Noria S.F., Brethauer S.A., Needleman B.J., Hsueh W., Reátegui E. (2025). Human Differentiated Adipocytes as Surrogate Mature Adipocytes for Adipocyte-Derived Extracellular Vesicle Analysis. Cells.

[108] Engin A.B. (2017). MicroRNA and Adipogenesis. Adv. Exp. Med. Biol..

[109] Lin W., Chen L., Meng W., Yang K., Wei S., Wei W., Chen J., Zhang L. (2022). C/EBPα promotes porcine pre-adipocyte proliferation and differentiation via mediating MSTRG.12568.2/FOXO3 trans-activation for STYX. Biochim. Biophys. Acta Mol. Cell Biol. Lipids.

[110] Shen S.M., Yu Y., Wu Z.X., Zheng Y., Chen G.Q., Wang L.S. (2011). Apoptosis-inducing factor is a target gene of C/EBPα and participates in adipocyte differentiation. FEBS Lett..

[111] Xu D., Zhuang S., Chen H., Jiang M., Jiang P., Wang Q., Wang X., Chen R., Tang H., Tang L. (2024). IL-33 regulates adipogenesis via Wnt/β-catenin/PPAR-γ signaling pathway in preadipocytes. J. Transl. Med..

[112] Zhao Y., Yao H., Liao Y., Jiang B., Li T., Chen J., Sheng Y., Yin M., Ye W., Yan Q. (2025). Selective PPARγ modulator alpinetin restores insulin sensitivity and protects from bone loss in type 2 diabetes. Phytomedicine.

[113] Li T., Li X., Meng H., Chen L., Meng F. (2020). ACSL1 affects Triglyceride Levels through the PPARγ Pathway. Int. J. Med. Sci..

[114] Kim J.B., Spiegelman B.M. (1996). ADD1/SREBP1 promotes adipocyte differentiation and gene expression linked to fatty acid metabolism. Genes Dev..

[115] Sha X., Lin J., Wu K., Lu J., Yu Z. (2025). The TRPV1-PKM2-SREBP1 axis maintains microglial lipid homeostasis in Alzheimer’s disease. Cell Death Dis..

[116] He Y., Zhang Y., Zhu S., Liu Y.F., Liu S., Xu Y.J. (2024). Monomethyl Branched-Chain Fatty Acids Suppress M1 Macrophage Polarization via FABP4/PPAR-γ Signaling Pathway. Mol. Nutr. Food Res..

[117] Tanaka M., Gohda T., Kamei N., Murakoshi M., Sato T., Kubota M., Sanuki M., Ishiwata E., Endo K., Suzuki Y. (2024). Associations between circulating levels of FABP4 and TNF receptors are more evident in patients with type 2 diabetes mellitus than in patients with type 1 diabetes mellitus. Endocr. Connect..

[118] Rajan P., Panchision D.M., Newell L.F., McKay R.D. (2003). BMPs signal alternately through a SMAD or FRAP-STAT pathway to regulate fate choice in CNS stem cells. J. Cell Biol..

[119] Liu Y., Sun Y., Lin X., Zhang D., Hu C., Liu J., Zhu Y., Gao A., Han H., Chai M. (2022). Perivascular adipose-derived exosomes reduce macrophage foam cell formation through miR-382-5p and the BMP4-PPARγ-ABCA1/ABCG1 pathways. Vasc. Pharmacol..

[120] Tang Y., Qian S.W., Wu M.Y., Wang J., Lu P., Li X., Huang H.Y., Guo L., Sun X., Xu C.J. (2016). BMP4 mediates the interplay between adipogenesis and angiogenesis during expansion of subcutaneous white adipose tissue. J. Mol. Cell Biol..

[121] Claussnitzer M., Dankel S.N., Kim K.H., Quon G., Meuleman W., Haugen C., Glunk V., Sousa I.S., Beaudry J.L., Puviindran V. (2015). FTO Obesity Variant Circuitry and Adipocyte Browning in Humans. N. Engl. J. Med..

[122] Jia G., Fu Y., Zhao X., Dai Q., Zheng G., Yang Y., Yi C., Lindahl T., Pan T., Yang Y.G. (2011). N6-methyladenosine in nuclear RNA is a major substrate of the obesity-associated FTO. Nat. Chem. Biol..

[123] Luo G., Ai Y., Zhu T., Li J., Ren Z. (2023). *FTO* promoted adipocyte differentiation by regulating *ADRB1* gene through m^6^A modification in Hycole rabbits. Anim. Biotechnol..

[124] Xu A., Zhang J., Zuo L., Yan H., Chen L., Zhao F., Fan F., Xu J., Zhang B., Zhang Y. (2022). FTO promotes multiple myeloma progression by posttranscriptional activation of HSF1 in an m^6^A-YTHDF2-dependent manner. Mol. Ther..

[125] Wu R., Liu Y., Yao Y., Zhao Y., Bi Z., Jiang Q., Liu Q., Cai M., Wang F., Wang Y. (2018). FTO regulates adipogenesis by controlling cell cycle progression via m^6^A-YTHDF2 dependent mechanism. Biochim. Biophys. Acta Mol. Cell Biol. Lipids.

[126] Yang Z., Ma X., Zhang D., Li B., Gao N., Li X., Mei C., Zan L. (2024). Bta-miR-330 promotes bovine intramuscular pre-adipocytes adipogenesis via targeting SESN3 to activate the Akt-mTOR signaling pathway. Int. J. Biol. Macromol..

[127] Guri Y., Colombi M., Dazert E., Hindupur S.K., Roszik J., Moes S., Jenoe P., Heim M.H., Riezman I., Riezman H. (2017). mTORC2 Promotes Tumorigenesis via Lipid Synthesis. Cancer Cell.

[128] Liu X., Guo B., Li Q., Nie J. (2024). mTOR in metabolic homeostasis and disease. Exp. Cell Res..

[129] Oishi Y., Manabe I., Tobe K., Tsushima K., Shindo T., Fujiu K., Nishimura G., Maemura K., Yamauchi T., Kubota N. (2005). Krüppel-like transcription factor KLF5 is a key regulator of adipocyte differentiation. Cell Metab..

[130] Xu Q., Lin Y., Wang Y., Bai W., Zhu J. (2020). Knockdown of KLF9 promotes the differentiation of both intramuscular and subcutaneous preadipocytes in goat. Biosci. Biotechnol. Biochem..

[131] Gulyaeva O., Dempersmier J., Sul H.S. (2019). Genetic and epigenetic control of adipose development. Biochim. Biophys. Acta Mol. Cell Biol. Lipids.

[132] Ibrahim H.I.M. (2022). Epigenetic Regulation of Obesity-Associated Type 2 Diabetes. Medicina.

[133] Lefterova M.I., Haakonsson A.K., Lazar M.A., Mandrup S. (2014). PPARγ and the Global Map of Adipogenesis and Beyond. Trends Endocrinol. Metab..

[134] Furuhashi M. (2019). Fatty Acid-Binding Protein 4 in Cardiovascular and Metabolic Diseases. J. Atheroscler. Thromb..

[135] Baboota R.K., Blüher M., Smith U. (2021). Emerging Role of Bone Morphogenetic Protein 4 in Metabolic Disorders. Diabetes.

[136] Shan Z., Fa W.H., Tian C.R., Yuan C.S., Jie N. (2022). Mitophagy and Mitochondrial Dynamics in Type 2 Diabetes Mellitus Treatment. Aging.

[137] Yang Z., Yu G.L., Zhu X., Peng T.H., Lv Y.C. (2021). Critical Roles of FTO-Mediated mRNA m^6^A Demethylation in Regulating Adipogenesis and Lipid Metabolism: Implications in Lipid Metabolic Disorders. Genes Dis..

[138] Liu Q., Zhao Y., Wu R., Jiang Q., Cai M., Bi Z., Liu Y., Yao Y., Feng J., Wang Y. (2019). ZFP217 Regulates Adipogenesis by Controlling Mitotic Clonal Expansion in a METTL3-m^6^A-Dependent Manner. RNA Biol..

[139] Cai M., Liu Q., Jiang Q., Wu R., Wang X., Wang Y. (2019). Loss of m^6^A on FAM134B Promotes Adipogenesis in Porcine Adipocytes Through m^6A-YTHDF2-Dependent Way. IUBMB Life.

[140] Bertolio R., Napoletano F., Mano M., Maurer-Stroh S., Fantuz M., Zannini A., Bicciato S., Sorrentino G., Del Sal G. (2019). Sterol Regulatory Element Binding Protein 1 Couples Mechanical Cues and Lipid Metabolism. Nat. Commun..

[141] Katagiri H. (2023). Inter-organ communication involved in metabolic regulation at the whole-body level. Inflamm. Regen..

[142] Gancheva S., Jelenik T., Álvarez-Hernández E., Roden M. (2018). Interorgan Metabolic Crosstalk in Human Insulin Resistance. Physiol. Rev..

[143] Sanches J.M., Zhao L.N., Salehi A., Wollheim C.B., Kaldis P. (2023). Pathophysiology of type 2 diabetes and the impact of altered metabolic interorgan crosstalk. FEBS J..

[144] Priest C., Tontonoz P. (2019). Inter-organ cross-talk in metabolic syndrome. Nat. Metab..

[145] Marra F., Bertolani C. (2009). Adipokines in liver diseases. Hepatology.

[146] Al-Mansoori L., Al-Jaber H., Prince M.S., Elrayess M.A. (2022). Role of Inflammatory Cytokines, Growth Factors and Adipokines in Adipogenesis and Insulin Resistance. Inflammation.

[147] Tilg H., Ianiro G., Gasbarrini A., Adolph T.E. (2025). Adipokines: Masterminds of metabolic inflammation. Nat. Rev. Immunol..

[148] Tsatsoulis A., Paschou S.A. (2020). Metabolically Healthy Obesity: Criteria, Epidemiology, Controversies, and Consequences. Curr. Obes. Rep..

[149] Fu J., Sun L., Mu Z., Xiu S. (2022). Free fatty acids are associated with muscle dysfunction in Chinese adults with type 2 diabetes. Endocrine.

[150] De Nardo W., Bayliss J., Elahee Doomun S.N., Lee O., Miotto P.M., Suriani N.D., Nie S., Leeming M., Miranda D.A., De Souza D.P. (2026). Effect of free fatty acids on TGF-β1 mediated fibrogenesis in hepatic stellate cells. Mol. Metab..

[151] Lee M.R., Yang H.J., Park K.I., Ma J.Y. (2019). *Lycopus lucidus* Turcz. ex Benth. Attenuates free fatty acid-induced steatosis in HepG2 cells and non-alcoholic fatty liver disease in high-fat diet-induced obese mice. Phytomedicine.

[152] Meex R.C.R., Blaak E.E., van Loon L.J.C. (2019). Lipotoxicity plays a key role in the development of both insulin resistance and muscle atrophy in patients with type 2 diabetes. Obes. Rev..

[153] An S.M., Cho S.H., Yoon J.C. (2023). Adipose Tissue and Metabolic Health. Diabetes Metab. J..

[154] Samuel V.T., Shulman G.I. (2012). Mechanisms for insulin resistance: Common threads and missing links. Cell.

[155] Havers T., Held S., Schönfelder M., Geisler S., Wackerhage H. (2025). Effects of Skeletal Muscle Hypertrophy on Fat Mass and Glucose Homeostasis in Humans and Animals: A Narrative Review with Systematic Literature Search. Sports Med..

[156] Paquin J., Tremblay R., Islam H., Riesco E., Marcotte-Chénard A., Dionne I.J. (2024). Resistance training, skeletal muscle hypertrophy, and glucose homeostasis: How related are they? A Systematic review and Meta-analysis. Appl. Physiol. Nutr. Metab..

[157] Merz K.E., Thurmond D.C. (2020). Role of Skeletal Muscle in Insulin Resistance and Glucose Uptake. Compr. Physiol..

[158] Severinsen M.C.K., Pedersen B.K. (2020). Muscle-Organ Crosstalk: The Emerging Roles of Myokines. Endocr. Rev..

[159] Bonaldo P., Sandri M. (2013). Cellular and molecular mechanisms of muscle atrophy. Dis. Model. Mech..

[160] da Silva Rosa S.C., Nayak N., Caymo A.M., Gordon J.W. (2020). Mechanisms of muscle insulin resistance and the cross-talk with liver and adipose tissue. Physiol. Rep..

[161] Fisher F.M., Maratos-Flier E. (2016). Understanding the Physiology of FGF21. Annu. Rev. Physiol..

[162] Dong H.N., Park S.Y., Le C.T., Choi D.H., Cho E.H. (2020). Irisin Regulates the Functions of Hepatic Stellate Cells. Endocrinol. Metab..

[163] Rossi J.F., Lu Z.Y., Jourdan M., Klein B. (2015). Interleukin-6 as a therapeutic target. Clin. Cancer Res..

[164] Dumond Bourie A., Potier J.B., Pinget M., Bouzakri K. (2023). Myokines: Crosstalk and Consequences on Liver Physiopathology. Nutrients.

[165] Krause M., De Vito G. (2023). Type 1 and Type 2 Diabetes Mellitus: Commonalities, Differences and the Importance of Exercise and Nutrition. Nutrients.

[166] Wang S.T., Zheng J., Peng H.W., Cai X.L., Pan X.T., Li H.Q., Hong Q.Z., Peng X.E. (2020). Physical activity intervention for non-diabetic patients with non-alcoholic fatty liver disease: A meta-analysis of randomized controlled trials. BMC Gastroenterol..

[167] Esteves J.V., Stanford K.I. (2024). Exercise as a tool to mitigate metabolic disease. Am. J. Physiol. Cell Physiol..

[168] Mancin L., Wu G.D., Paoli A. (2023). Gut microbiota-bile acid-skeletal muscle axis. Trends Microbiol..

[169] Fusco W., Lorenzo M.B., Cintoni M., Porcari S., Rinninella E., Kaitsas F., Lener E., Mele M.C., Gasbarrini A., Collado M.C. (2023). Short-Chain Fatty-Acid-Producing Bacteria: Key Components of the Human Gut Microbiota. Nutrients.

[170] Yang W., Cong Y. (2021). Gut microbiota-derived metabolites in the regulation of host immune responses and immune-related inflammatory diseases. Cell. Mol. Immunol..

[171] Tang H., Li K., Shi Z., Wu J. (2025). G-Protein-Coupled Receptors in Chronic Kidney Disease Induced by Hypertension and Diabetes. Cells.

[172] Han B., Lv X., Liu G., Li S., Fan J., Chen L., Huang Z., Lin G., Xu X., Huang Z. (2023). Gut microbiota-related bile acid metabolism-FXR/TGR5 axis impacts the response to anti-α4β7-integrin therapy in humanized mice with colitis. Gut Microbes.

[173] Wang Q., Lin H., Shen C., Zhang M., Wang X., Yuan M., Yuan M., Jia S., Cao Z., Wu C. (2023). Gut microbiota regulates postprandial GLP-1 response via ileal bile acid-TGR5 signaling. Gut Microbes.

[174] Anhê F.F., Barra N.G., Cavallari J.F., Henriksbo B.D., Schertzer J.D. (2021). Metabolic endotoxemia is dictated by the type of lipopolysaccharide. Cell Rep..

[175] Candelli M., Franza L., Pignataro G., Ojetti V., Covino M., Piccioni A., Gasbarrini A., Franceschi F. (2021). Interaction between Lipopolysaccharide and Gut Microbiota in Inflammatory Bowel Diseases. Int. J. Mol. Sci..

[176] Miyachi Y., Tsuchiya K., Ogawa Y. (2016). Lifestyle-related diseases and an inter-organ metabolic network. Clin. Calcium.

[177] Santos L. (2022). The impact of nutrition and lifestyle modification on health. Eur. J. Intern. Med..

[178] Yang J., Zhang Q., Zhao W., Ye B., Li S., Zhang Z., Ju J., He J., Xia M., Xiong T. (2024). Associations of traditional healthy lifestyle and sleep quality with metabolic dysfunction-associated fatty liver disease: Two population-based studies. Nutr. Diabetes.

[179] Jiao H., Kalsbeek A., Yi C.X. (2024). Microglia, circadian rhythm and lifestyle factors. Neuropharmacology.

[180] Henson J., De Craemer M., Yates T. (2023). Sedentary behaviour and disease risk. BMC Public Health.

[181] Liang Y.Y., He Y., Huang P., Feng H., Li H., Ai S., Du J., Xue H., Liu Y., Zhang J. (2025). Accelerometer-measured physical activity, sedentary behavior, and incidence of macrovascular and microvascular events in individuals with type 2 diabetes mellitus and prediabetes. J. Sport Health Sci..

[182] Strasser B., Wolters M., Weyh C., Krüger K., Ticinesi A. (2021). The Effects of Lifestyle and Diet on Gut Microbiota Composition, Inflammation and Muscle Performance in Our Aging Society. Nutrients.

[183] Thyfault J.P., Bergouignan A. (2020). Exercise and metabolic health: Beyond skeletal muscle. Diabetologia.

[184] Hawley J.A., Forster S.C., Giles E.M. (2025). Exercise, the Gut Microbiome and Gastrointestinal Diseases: Therapeutic Impact and Molecular Mechanisms. Gastroenterology.

[185] Karolkiewicz J., Krzywicka M., Szulińska M., Musialik K., Musiałowska D., Zieliński J., Bilska A., Ratajczak M. (2024). Effects of a Circuit Training Program on Myokine Levels in Insulin-Resistant Women: A Randomised Controlled Trial. J. Diabetes Res..

[186] Bagheri R., Ashtary-Larky D., Elliott B.T., Willoughby D.S., Kargarfard M., Alipour M., Lamuchi-Deli N., Kooti W., Asbaghi O., Wong A. (2023). The effects of gradual vs. rapid weight loss on serum concentrations of myokines and body composition in overweight and obese females. Arch. Physiol. Biochem..

[187] Calçada D., Vianello D., Giampieri E., Sala C., Castellani G., de Graaf A., Kremer B., van Ommen B., Feskens E., Santoro A. (2014). The role of low-grade inflammation and metabolic flexibility in aging and nutritional modulation thereof: A systems biology approach. Mech. Ageing Dev..

[188] Smith R.L., Soeters M.R., Wüst R.C.I., Houtkooper R.H. (2018). Metabolic Flexibility as an Adaptation to Energy Resources and Requirements in Health and Disease. Endocr. Rev..

[189] Tian H., Li H., Zhang X., Liu H., Huang L., Yu H., Wu J., Cao Y., Peng L., García-Ramos A. (2024). Non-pharmacological treatment strategies for anthropometric, physical capacity and physiological indicators among sarcopenic obesity patients: A systematic review of rigorous randomized controlled trials. Age Ageing.

[190] Sylow L., Kleinert M., Richter E.A., Jensen T.E. (2017). Exercise-stimulated glucose uptake—Regulation and implications for glycaemic control. Nat. Rev. Endocrinol..

[191] Da Rosa P.C., Bertomeu J.B., Royes L.F.F., Osiecki R. (2023). The physical exercise-induced oxidative/inflammatory response in peripheral blood mononuclear cells: Signaling cellular energetic stress situations. Life Sci..

[192] Egan B., Sharples A.-P. (2023). Molecular responses to acute exercise and their relevance for adaptations in skeletal muscle to exercise training. Physiol. Rev..

[193] Koh H.J., Hirshman M.F., He H., Li Y., Manabe Y., Balschi J.A., Goodyear L.J. (2007). Adrenaline is a critical mediator of acute exercise-induced AMP-activated protein kinase activation in adipocytes. Biochem. J..

[194] Yue Y., Zhang C., Zhang X., Zhang S., Liu Q., Hu F., Lv X., Li H., Yang J., Wang X. (2020). An AMPK/Axin1-Rac1 signaling pathway mediates contraction-regulated glucose uptake in skeletal muscle cells. Am. J. Physiol. Endocrinol. Metab..

[195] Flores-Opazo M., McGee S.L., Hargreaves M. (2020). Exercise and GLUT4. Exerc. Sport. Sci. Rev..

[196] Mingzheng X., You W. (2025). AMPK/mTOR balance during exercise: Implications for insulin resistance in aging muscle. Mol. Cell. Biochem..

[197] Yao Z., Jiang Z., Liu X., Zhang L., Guo S., Chen Y., Luo L., Ma S., Wang P., Shyh-Chang N. (2025). Muscle Organoids Reveal Exercise-Like Contractions Rapidly Promote Muscle Health Via Lamtor1’s Signaling to Both AMPK and mTOR. Adv. Sci..

[198] Wrann C.D., White J.P., Salogiannnis J., Laznik-Bogoslavski D., Wu J., Ma D., Lin J.D., Greenberg M.E., Spiegelman B.M. (2013). Exercise induces hippocampal BDNF through a PGC-1α/FNDC5 pathway. Cell Metab..

[199] Zheng L., Rao Z., Wu J., Ma X., Jiang Z., Xiao W. (2024). Resistance Exercise Improves Glycolipid Metabolism and Mitochondrial Biogenesis in Skeletal Muscle of T2DM Mice via miR-30d-5p/SIRT1/PGC-1α Axis. Int. J. Mol. Sci..

[200] Pérez-López A., Gonzalo-Encabo P., Pérez-Köhler B., García-Honduvilla N., Valadés D. (2022). Circulating myokines IL-6, IL-15 and FGF21 response to training is altered by exercise type but not by menopause in women with obesity. Eur. J. Sport. Sci..

[201] Pan Y., Mary Peter R., Chou P., Dave P.D., Xu J., Shanner A., Sarwar M.S., Kong A.N. (2025). Cancer-specific Regulation of Metabolic and Epigenetic Pathways by Dietary Phytochemicals. Pharm. Res..

[202] Pesta D., Jordan J. (2022). Macronutrient composition and metabolic regulation: Do our islets care what we eat?. Acta Physiol..

[203] Li Y., Cheng Y., Zhou Y., Du H., Zhang C., Zhao Z., Chen Y., Zhou Z., Mei J., Wu W. (2022). High fat diet-induced obesity leads to depressive and anxiety-like behaviors in mice via AMPK/mTOR-mediated autophagy. Exp. Neurol..

[204] Poznyak A., Grechko A.V., Poggio P., Myasoedova V.A., Alfieri V., Orekhov A.N. (2020). The Diabetes Mellitus-Atherosclerosis Connection: The Role of Lipid and Glucose Metabolism and Chronic Inflammation. Int. J. Mol. Sci..

[205] Esch N., Jo S., Moore M., Alejandro E.U. (2020). Nutrient Sensor mTOR and OGT: Orchestrators of Organelle Homeostasis in Pancreatic *β*-Cells. J. Diabetes Res..

[206] Arrieta-Cruz I., Torres-Ávila B.S., Martínez-Coria H., López-Valdés H.E., Gutiérrez-Juárez R. (2022). Diet-Induced Metabolic Dysfunction of Hypothalamic Nutrient Sensing in Rodents. Int. J. Mol. Sci..

[207] Barber T.M., Kabisch S., Pfeiffer A.F.H., Weickert M.O. (2020). The Health Benefits of Dietary Fibre. Nutrients.

[208] Mantle D., Hargreaves I.P. (2022). Mitochondrial Dysfunction and Neurodegenerative Disorders: Role of Nutritional Supplementation. Int. J. Mol. Sci..

[209] Lin C.C., Tsweng G.J., Lee C.F., Chen B.H., Huang Y.L. (2016). Magnesium, zinc, and chromium levels in children, adolescents, and young adults with type 1 diabetes. Clin. Nutr..

[210] Koekkoek K.W.A., Berger M.M. (2023). An update on essential micronutrients in critical illness. Curr. Opin. Crit. Care.

[211] Islam M.N., Nabekura H., Ueno H., Nishida T., Nanashima A., Sakoda H., Zhang W., Nakazato M. (2024). Liver-expressed antimicrobial peptide 2 is a hepatokine regulated by ghrelin, nutrients, and body weight. Sci. Rep..

[212] Smith H.A., Betts J.A. (2022). Nutrient timing and metabolic regulation. J. Physiol..

[213] Palabiyik A.A., Palabiyik E. (2025). Pharmacological approaches to enhance mitochondrial biogenesis: Focus on PGC-1A, AMPK, and SIRT1 in cellular health. Mol. Biol. Rep..

[214] González-Casanova J.E., Navarro-Márquez M., Sáez-Tamayo T., Angarita L., Durán-Agüero S., Fuentes-Barría H., Bermúdez V., Rojas-Gómez D.M. (2025). New Perspectives on the Molecular Action of Metformin in the Context of Cellular Transduction and Adipogenesis. Int. J. Mol. Sci..

[215] Ahn C., Hevener A.L., Goodyear L.J., Bodine S.C., Esser K.A., Seldin M.M., Sparks L.M. (2025). Exercise training remodels inter-organ endocrine networks. Mol. Metab..

[216] Verboven K., Vechetti I.J. (2023). Editorial: Inter-organ crosstalk during exercise in health and disease: Extracellular vesicles as new kids on the block. Front. Physiol..

[217] Garruti G., Baj J., Cignarelli A., Perrini S., Giorgino F. (2023). Hepatokines, bile acids and ketone bodies are novel Hormones regulating energy homeostasis. Front. Endocrinol..

[218] Giovanini L., Wanionok N., Perello M., Cornejo M.P. (2025). Brain-acting hepatokines: Its impact on energy balance and metabolism. Front. Neurosci..

[219] He M., Wei W., Zhang Y., Xiang Z., Peng D., Kasimumali A., Rong S. (2024). Gut microbial metabolites SCFAs and chronic kidney disease. J. Transl. Med..

[220] Mukhopadhya I., Louis P. (2025). Gut microbiota-derived short-chain fatty acids and their role in human health and disease. Nat. Rev. Microbiol..

[221] Angione C. (2019). Human Systems Biology and Metabolic Modelling: A Review-From Disease Metabolism to Precision Medicine. Biomed Res. Int..

[222] Mardinoglu A., Nielsen J. (2012). Systems medicine and metabolic modelling. J. Intern. Med..

[223] Agrawal P., Kaur J., Singh J., Rasane P., Sharma K., Bhadariya V., Kaur S., Kumar V. (2024). Genetics, Nutrition, and Health: A New Frontier in Disease Prevention. J. Am. Nutr. Assoc..

[224] Bowman M.A., Seehusen D.A., Neale A.V. (2019). Implementing Practice Changes in Family Medicine to Enhance Care and Prevent Disease Progression. J. Am. Board Fam. Med..

[225] Yip H.F., Li Z., Zhang L., Lyu A. (2025). Large Language Models in Integrative Medicine: Progress, Challenges, and Opportunities. J. Evid. Based Med..

[226] Kolb H., Martin S. (2017). Environmental/lifestyle factors in the pathogenesis and prevention of type 2 diabetes. BMC Med..

[227] Balducci S., Haxhi J., Sacchetti M., Orlando G., Cardelli P., Vitale M., Mattia L., Iacobini C., Bollanti L., Conti F. (2022). Italian Diabetes and Exercise Study 2 (IDES_2) Investigators. Relationships of Changes in Physical Activity and Sedentary Behavior With Changes in Physical Fitness and Cardiometabolic Risk Profile in Individuals With Type 2 Diabetes: The Italian Diabetes and Exercise Study 2 (IDES_2). Diabetes Care.

[228] Gram-Kampmann E.M., Olsen M.H., Beck-Nielsen H. (2016). Low-carbohydrate diet for patients with Type 2 diabetes. Ugeskr. Laeger.

[229] Park J.S., Holloszy J.O., Kim K., Koh J.H. (2020). Exercise Training-Induced PPARβ Increases PGC-1α Protein Stability and Improves Insulin-Induced Glucose Uptake in Rodent Muscles. Nutrients.

[230] Olayaki L.A., Okesina K.B., Jesubowale J.D., Ajibare A.J., Odetayo A.F. (2023). Orange Peel Extract and Physical Exercise Synergistically Ameliorate Type 2 Diabetes Mellitus-Induced Dysmetabolism by Upregulating GLUT4 Concentration in Male Wistar Rats. J. Med. Food.

[231] Petroni M.L., Brodosi L., Marchignoli F., Sasdelli A.S., Caraceni P., Marchesini G., Ravaioli F. (2021). Nutrition in Patients with Type 2 Diabetes: Present Knowledge and Remaining Challenges. Nutrients.

[232] Bruner K.R., Byington I.R., Marx T.J., Vasileva A., Fletcher T., Ghimire S., Zappia I.J., Shaju Y., Zeng J., Wachsmuth H.R. (2025). Glucagon receptor signaling is indispensable for the healthspan effects of caloric restriction in aging male mice. GeroScience.

[233] Weickert M.O., Pfeiffer A.F.H. (2018). Impact of Dietary Fiber Consumption on Insulin Resistance and the Prevention of Type 2 Diabetes. J. Nutr..

[234] Chen L., Liu B., Ren L., Du H., Fei C., Qian C., Li B., Zhang R., Liu H., Li Z. (2023). High-fiber diet ameliorates gut microbiota, serum metabolism and emotional mood in type 2 diabetes patients. Front. Cell. Infect. Microbiol..

[235] Delrue C., Speeckaert R., Speeckaert M.M. (2025). The role of intermittent fasting and ketogenic diet in metabolic syndrome and type 2 diabetes. Acta Clin. Belg..

